# Oligomer formation of SARS-CoV-2 ORF8 through 73YIDI76 motifs regulates immune response and non-infusion antiviral interactions

**DOI:** 10.3389/fmolb.2023.1270511

**Published:** 2023-11-29

**Authors:** Mohammad Assadizadeh, Maryam Azimzadeh Irani

**Affiliations:** Faculty of Life Sciences and Biotechnology, Shahid Beheshti University, Tehran, Iran

**Keywords:** SARS-CoV-2, ORF8, oligomerization, molecular modeling, molecular dynamics, B-cell, MHC-I, non-infusion antiviral

## Abstract

**Introduction:** Open Reading Frame 8 (ORF8) is a 121 amino acid length SARS-CoV-2 specific accessory protein that plays crucial roles in viral infectivity, and pathogenesis. Current SARS-CoV-2 treatments focus on spike or RNA-dependent RNA polymerase proteins. Hence, directing attention to ORF8 yields substantial benefits for innovative non-infusional therapeutics. Functional ORF8 is proposed to form oligomers via a crystallographic contact centered by 73YIDI76 motifs.

**Methods:** Hence, the structure and atomistic interactions of trimeric and tetrameric ORF8 oligomeric forms were modeled by means of thorough molecular modeling and molecular dynamics simulations.

**Results:** Results show that trimeric and tetrameric oligomers are stabilized by the interaction of β4-β5 (47-83) loops. 73YIDI76 motifs are involved in obtaining the oligomerization interfaces. It is shown that the tetramers which resemble a doughnut-like construction are the most stabilized oligomeric forms. Where four β4-β5 loops form the interfaces between two dimers. Each monomer links to two others through β4-β5 loops and a covalent Cys20-Cys20 bridge. Epitope mapping, binding site predictions, and solvent-accessible surface area analyses of different ORF8 forms show that the B-cell, MHC-I, and drug epitopes stay exposed in oligomeric forms.

**Discussion:** Approving that the viral infectivity is expanded upon ORF8 oligomerization and the regions involved in oligomerization can be considered as therapeutic targets.

## 1 Introduction

Coronaviruses are enveloped single-stranded RNA viruses that were spotlighted throughout the coronavirus disease 2019 (COVID-19) pandemic caused by severe acute respiratory syndrome coronavirus 2 (SARS-CoV-2)) (Lai et al., 2020; Sohrabi et al., 2020). SARS-CoV, MERS, and SARS-CoV-2 are species belonging to the genus Betacoronavirus that have led to serious human diseases (Gong and Bao, 2018; Cui et al., 2019; Kandeel et al., 2020). By 31 July 2023, SARS-CoV-2 had caused more than 6.9 million fatalities and 768.5 million COVID-19 cases.

SARS-CoV-2 RNA genome is composed of 12 open reading frames (ORFs) that encode four structural proteins, 16 nonstructural proteins (Nsps), and 11 accessory proteins (Kim et al., 2020; V’kovski et al., 2021; Yan et al., 2022). Structural proteins are required for virion assembly, while Nsps are involved in transcription and viral replication (Snijder et al., 2016). Although their precise functions are still unclear, accessory proteins have the ability to influence the immune system by interacting with the host cell’s molecular network (Redondo et al., 2021; Zandi et al., 2022). One of SARS-CoV-2 accessory proteins known as ORF8 is thought to have a substantial correlation with the pathogenesis of the virus (Vinjamuri et al., 2022).

The structural characteristics, and significant interactions of ORF8 allow it to suppress and evade the immune system (Vinjamuri et al., 2022; Kim et al., 2023). As a result of a 382-nucleotide deletion in the ORF8 gene, patients experienced milder infections (Su et al., 2020; Young et al., 2020).

Experimental studies identified 47 human proteins as ORF8 interacting partners (Gordon et al., 2020). Subsequently, ORF8 was statistically introduced as the most significant protein of virus, along with the membrane (M) protein and Nsp7, in terms of their connections with human protein networks (Díaz, 2020).

ORF8 interacting partners are associated with virus pathogenesis, ER stress, and antiviral immune responses, including disruption of the interferon type I signaling pathway (Kumar et al., 2020; Li et al., 2020; Fahmi et al., 2021; Rashid et al., 2021; Stukalov et al., 2021; Liu et al., 2022; Matsuoka et al., 2022; Takatsuka et al., 2022; Wang et al., 2023).

ORF8 mimics various immune molecules, such as Interleukin-17, resulting in IL-17 receptors A and C activation that causes a quite strong inflammatory response (Tan et al., 2020; Valcarcel et al., 2021; Venkatesan et al., 2022; Wu et al., 2022).

In addition to ORF8 involvement in many other influential pathways, ORF8 leads to immune evasion by interacting directly with major histocompatibility complex class Ι (MHC-1), and downregulating its expression (Zhang et al., 2020; Zhang et al., 2021).

In the structure of 121-amino acid ORF8, an Ig-like domain follows a 15-amino acid signal peptide at the N-terminus. Reducing the three intramolecular disulfide bonds that hold the eight antiparallel ß-strands together that the Ig-like domain folds into has been shown to have a negligible impact on the system’s overall characteristics (Cheng and Peng, 2022). ORF8 structure is experimentally obtained as a covalently bonded dimer by X-ray crystallography (Flower et al., 2021). At the center of covalent interface, two Cys20 from monomers create an interchain disulfide bond. Other residues, such as Val117, Arg115, and Asp119 also play a role in stabilizing the covalent interface. Furthermore, the loops surrounding the interface contribute to its stability through hydrogen bondings (Chen et al., 2021; Flower et al., 2021). The key residues in the dimerization interface could be changed via the mutations, leading to dimer instability (Ahmadi et al., 2022).

Many studies are predicated on the idea that crystallographic contacts represent true macromolecular interactions that may be used in docking investigations (Krissinel, 2010). The X-ray crystallographic data indicate that the ORF8 dimer contact may function as a non-covalent oligomerization interface (Flower et al., 2021). A loop area between β4 and β5 positioned at 47-83 distinct region of ORF8 contains a unique 73YIDI76 motif, two of which form the crystallographic contact by being situated adjacent to one another as parallel strands. Tyr73 of the 73YIDI76 motifs interacts hydrophobically with Leu95, Ile58, Val49, and Pro56 of the same chain (Flower et al., 2021).

This unique β4-β5 loop is exposed at the surface of the dimer and exhibits high flexibility in MD studies (Chaudhari et al., 2023; Islam et al., 2023; Selvaraj et al., 2023). This conformational flexibility suggests the ability of the loop to adopt multiple conformations and potentially interact with a wide range of protein or ligand partners (Dyson and Wright, 2018; Mishra et al., 2020; Chen et al., 2021; Hu et al., 2023).

It is consistently predicted in past studies that the MHC-I epitope of ORF8 is located at positions 39–42, 104–107, and 110–112 (Can et al., 2020; Cheng and Peng, 2022; Chaudhari et al., 2023). Despite ORF8 dimerization, the reductions of disulfide bonds have no significant influence on its MHC-I binding site (Cheng and Peng, 2022; Chaudhari et al., 2023).

Since homodimers of ORF8 can be released from infected cells, they can trigger an intense immune response. Experimentally proposed linear B-cell epitopes for ORF8 are positioned at the 33-42 region (aligned with a part of the MHC-I binding site), and the β4-β5 loop (Wang et al., 2021).

Another significant structural element is Deep Groove Between the Monomers (DGBM) that is formed by 51AR52 and 92EPKL95 assembling on the ORF8 dimeric covalent interface. The charged residues in DGBM, and their structural arrangement make DGMB a possible hotspot binding site in the form of a polar pit.

A structural representation of ORF8 can be found in the Supplementary Materials.

SARS-CoV-2 unique ORF8 is considered to be able to form functional multimeric assemblies, especially tetramers, which is not reported in SARS-CoV (Flower et al., 2021; Matsuoka et al., 2022; Kumar et al., 2023). ORF8 oligomers are proposed to lead to ER stress, suppression, and evasion of the immune system (Flower et al., 2021; Liu et al., 2022; Matsuoka et al., 2022; Kumar et al., 2023).

Furthermore, ORF8 is a suitable candidate for non-infusion protein inhibitor design since it plays a critical role in virus-cell molecular interaction network (Prussia et al., 2011; Díaz, 2020; Valcarcel et al., 2021; Vinjamuri et al., 2022; Yan et al., 2022; Lin et al., 2023). The current COVID-19 treatments which focus on the virus spike, and RNA-dependent RNA polymerase (RdRp) are practical as a short-term solution to the pandemic, whereas further ORF8-targeted drug design can lead to the development of more efficient antiviral therapeutics. As a consequence, concentrating on ORF8’s oligomerized structure may provide more precise findings and illuminate a variety of features of ORF8 function. In spite of the significance of the problem, not much is known about the interactions and structures of ORF8 oligomers. This is the first attempt to target the formation, and drug-binding affinity of oligomeric assemblies of the promising non-infusion protein candidate ORF8 at the atomic level. In this regard, the ORF8 trimer and tetramer structures were modeled and the atomistic interactions formed in each assembly were investigated. Consequently, molecular dynamics simulations were performed to study the dynamics and stability of the oligomeric systems. Finally, the important binding sites and drug target hotspots were examined in oligomeric forms to assess the effect of oligomerization on these regions.

## 2 Methods

### 2.1 PDB file preparations

The 3D structure of ORF8 dimer was obtained from the Protein Data Bank with PDB ID 7jtl (Berman et al., 2000; Berman et al., 2003; Burley et al., 2021; Flower et al., 2021). Several residues were not located in each chain of the 7jtl, including Ser15, Asn16, Ala17, Ala65, and Gly66 in chain A and Asn16, Ala17, Gly66, Ser67, and Lys68 in chain B. Modeller 10.2 was used to model the missing residues, and the complete structure was created by aligning the full-length sequence with the sequence without missing residues, wherein each missing residue was defined as a gap (Fiser et al., 2000; Marti-Renom et al., 2002; Eswar et al., 2003; Fiser and Sali, 2003). The 7jtl was used as the modeling template. Energy minimization was then performed on the resulting structure by chimera 1.16 using AMBER ff14SB force field (Pettersen et al., 2004; Maier et al., 2015; Tian et al., 2020). The minimization was executed by two methods, first the steepest descent method in 1000 steps and then the conjugate gradient in 2000 steps. Each step size was defined as 0.02 Å. Using PROCHECK and Pro-SA, the derived dimer’s model quality was evaluated (Laskowski et al., 1996; Hunter, 2007; Wiederstein and Sippl, 2007; Saini et al., 2019; Saini et al., 2021). The full-length dimer model has excellent statistical fittingness and stereochemical quality. Energy minimization and model quality assessment results are presented in the Supplementary Materials (Supplementary Figures S2, S3). Molecular docking was used to predict the structure of ORF8 trimers, and tetramers from the energy-minimized full-length structure.

### 2.2 Molecular docking

Three sets of molecular dockings were performed by HADDOCK2.4 using default parameters (Dominguez et al., 2003; van Zundert et al., 2016). HADDOCK score, which is computed by a weighted sum of multiple energy terms, and Buried Surface Area (BSA) between the docked components, was used to rank the generated clusters.

ORF8 trimers were made by docking the monomer to the dimer. 73YIDI76 residues were selected in the monomer, and one of the dimer’s monomers as active residues directly involved in the docking process. Active residues were chosen based on the plausible oligomerization site that was suggested in X-ray experiments (Krissinel, 2010; Flower et al., 2021).

The tetramers were constructed by combining two ORF8 dimers using two different methods. In the first method, the residues 73YIDI76 were identified as active residues in one monomer of each dimer (refer to Table 2). In the second method, these residues were designated as active residues in all four monomers (see to Table 3).

### 2.3 Structural visualization and atomistic interaction investigation

Structures were visualized, and analyzed by PyMOL2 software. Interface residues between monomers were identified by subtracting the complex surface areas from the surface areas of separate chains with a cutoff of 1.0 Å^2^.

The atomistic interactions of interface residues were visualized in two-dimensional plots using the DIMPLOT program from the LigPlus software (Laskowski and Swindells, 2011).

### 2.4 Molecular dynamics simulation

Structural stability and dynamics of the tetrameric, and trimeric forms of ORF8 were investigated by performing molecular dynamics simulations using GROMACS 2021.4 software (Abraham et al., 2015; Páll et al., 2015).

The modeled trimer structure, and the best structure of the second set of tetramer docking were used as starting points for the simulations. The systems were prepared by generating topologies using OPLS-AA/M force field (Kaminski et al., 2001; Robertson et al., 2015; Dodda et al., 2017).

Afterward, we solvated the system in a Rectangular cuboid box of water molecules, ensuring a minimum distance of 10 Å between the proteins and the box edges. A model of transferable intermolecular potential with three points (TIP3P) was used to simulate the water molecules. Counter ions were added to neutralize systems (20 and 15 Na ions for tetramer and trimer respectively).

A two-step approach of steepest descent and the conjugate gradient was used for the energy minimization of systems. Firstly, the steepest descent algorithm was involved with a 50,000 maximum number of steps, maximum force tolerance of 500 kJ/mol/nm, and energy step size of 0.01 nm. Consequently, the conjugate gradient algorithm was applied for another maximum of 50,000 steps with a maximum force tolerance of 100 kJ/mol/nm and an energy step size of 0.01 nm until convergence was achieved (Schlick, 2010; Azimzadeh Irani, 2020).

For all the simulations, the LINCS algorithm was used to constrain bond lengths, the Periodic Boundary Conditions (PBC) to simulate an infinite system, a cutoff of 10 Å for the calculation of short-range nonbonded interactions, and Particle Mesh Ewald (PME) for long-range electrostatic interactions (Bondi, 1964; Darden et al., 1993; Hess et al., 1997).

NVT ensemble is used at 310 K to maintain a constant temperature through a 100 ps simulation with a time step of 1 fs. The systems were heated up to 310 K from the starting temperature of 0 K using an annealing method that gradually increased temperatures during 4 steps and then the temperature of the system was maintained at 310 K using the V-rescale temperature coupling algorithm (a modified Berendsen thermostat) (Berendsen et al., 1984). Position restraints are applied to the proteins to prevent large fluctuations in their structure.

After the NVT equilibration, the systems were equilibrated further by a 250-ps simulation under an NPT ensemble at 310 K and 1 bar with a time step of 1 fs. V-rescale and Berendsen methods were employed for temperature, and pressure coupling, respectively. (energy minimization and system equilibration results in Supplementary Materials).

Ultimately, production runs were conducted in three replicates of 100 ns with a 1 fs time step for each of the trimeric and tetrameric models all of which performed under the NPT ensemble, with V-rescale thermostat and Parrinello-Rahman pressure coupling algorithm maintaining 310 K and 1 bar respectively. Coordinates were saved every 10 ps for analysis.

### 2.5 Analyses of MD simulations results

To prepare trajectories, the GROMACS 2021.4 trjconv module was used. The protein systems were centered in each trajectory, fitted rotationally-transitionally to the initial conformation using the Least-squares fitting technique, and then PBC treatment was used for further computations.

The sasa package of GROMACS 2021.4 was used to calculate the solvent-accessible surface area (SASA) of covalent interface, DGBM, β4-β5 loops (the two free loops of dimers, and just the engaged loops for trimers and tetramers), and MHC-I binding sites. The distance package was utilized to calculate the distances between the centers of mass (COM) of non-interacting A-C and B-D monomers over time. The cluster package was employed to conduct clustering and determine the central structure for each cluster, using a defined cutoff of 3 Å.

Bio3D package in R was utilized to provide RMSD, RMSF, principal component analysis (PCA), and dynamic cross-correlation matrix (DCCM) of the Cα atoms (Grant et al., 2006).

All provided data was visualized in R (Bio3D and ggplot2 packages) and Python language using the matplotlib package (Hunter, 2007; Wickham, 2016).

Visual molecular dynamics (VMD) software was employed for secondary structure timeline analyses for 300 frames over 100 ns (Humphrey et al., 1996).

### 2.6 Binding site prediction

The parameter-free geometric deep learning tool PeSTo was used to conduct high-confidence structural-based prediction of possible binding sites for several structures, including minimized ORF8 dimer, trimer, and tetramer (Krapp et al., 2023). Interfaces involved in protein-protein and small-molecule interactions were compared in the various complexes of ORF8.

The dimer of ORF8, and the average structure from the five most populated clusters within each trajectory of trimer and tetramer (in total 15 structures for trimer, 15 structures for tetramer) were submitted to the Ellipro server to B-cell epitope prediction (Ponomarenko et al., 2008). Ellipro utilizes a combination of surface accessibility, flexibility, and antigenicity calculations to identify potential B-cell epitopes. Maximum score and maximum distance are set at 0.6 and 4 Å, respectively. The results of different assemblies were visualized and compared to gain insights into the impact of oligomerization on B-cell epitopes.

## 3 Results

### 3.1 Modeling of the trimeric construct

From the docking outputs, 195 provided trimers were bunched into three clusters. Cluster 1 is the most statistically reliable one, containing 87.2 percent of the docked structures. Compared to the other clusters, cluster 1 has a higher BSA of 1565.7 ± 61.3 Å^2^. This value is closer to that of the suggested non-covalent interface. The root-mean-square deviation (RMSD) values of trimer structures vary between 0 and 4 Å, with most structures populating in the 0 to 1 Å. The interface van der Waals energy of constructed structures varied from −20 to −75 kcal/mol and constituents of cluster 1 show stronger van der Waals interactions. Moreover, the interface electrostatic energy of assembled structures ranged from −20 to −80 kcal/mol, and Clusters 1, 2, and 3 have mean electrostatic energies of −62.6, −55.0, and −32.7 kcal/mol, respectively. Cluster 1 structures are typically more stable due to lower RMSD and interaction energy values (Table 1).

**TABLE 1:** Statistics of trimer docking results. The clusters in the columns are sorted from left to right based on the HADDOCK score. A more negative HADDOCK score and Z-score indicate better energy and statistical significance. Cluster 1 is the most populated and reliable.

Cluster number	1	2	3
HADDOCK score	−101.6 ± 2.0	−72.3 ± 6.4	−56.4 ± 3.5
Cluster size	170	21	4
RMSD from the overall lowest energy structure	1.4 ± 1.0	1.4 ± 0.4	4.7 ± 0.4
Van der Waals energy	−71.8 ± 2.1	−46.5 ± 5.0	−38.5 ± 3.9
Electrostatic energy	−62.6 ± 9.7	−55.0 ± 5.6	−32.7 ± 7.6
Desolvation energy	−17.9 ± 1.7	−16.3 ± 1.2	−13.1 ± 1.6
Restraints violation energy	6.3 ± 3.8	14.2 ± 6.7	17.4 ± 14.4
Buried Surface Area	1565.7 ± 61.3	1186.2 ± 51.2	1057.4 ± 90.6
Z-Score	−1.3	0.2	1.1

**FIGURE 1:**
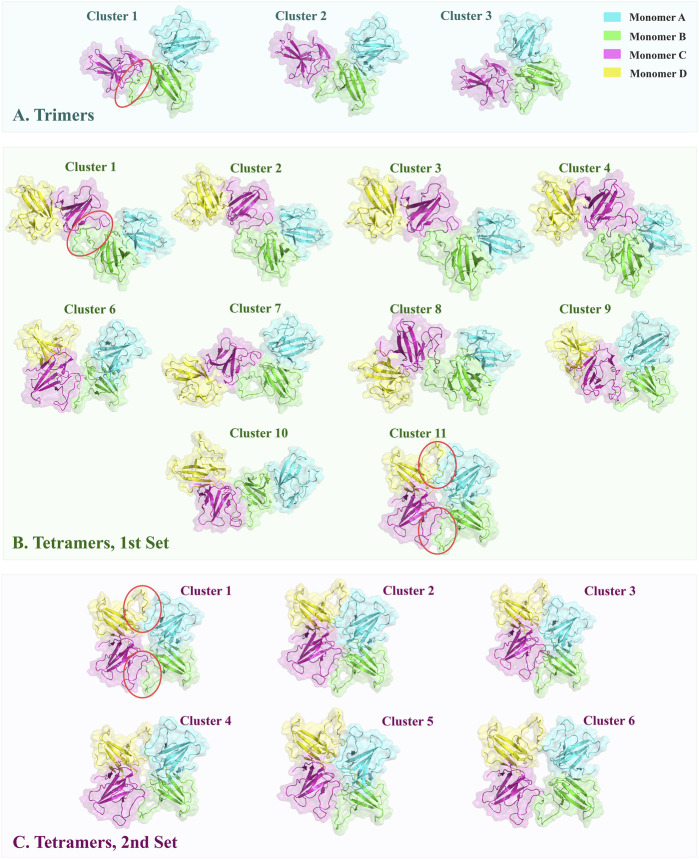
The top-ranked oligomeric structures of clusters obtained from docking were shown in cartoon style with transparent surfaces. The interfaces formed through docking are marked by red circles. **(A)** ORF8 trimers were constructed by docking the monomer C on the dimers. The typical arrangement is conserved via the three clusters. **(B)** The first set of tetramers was modeled by defining one docking site on each dimer. Cluster 11 has a distinctive arrangement of monomers, forming a doughnut-like construction with two interfaces between dimers. **(C)** In the second set of tetramers, two docking sites were specified on each dimer. All resulting structures resembled cluster 11 in the first set.

**FIGURE 2:**
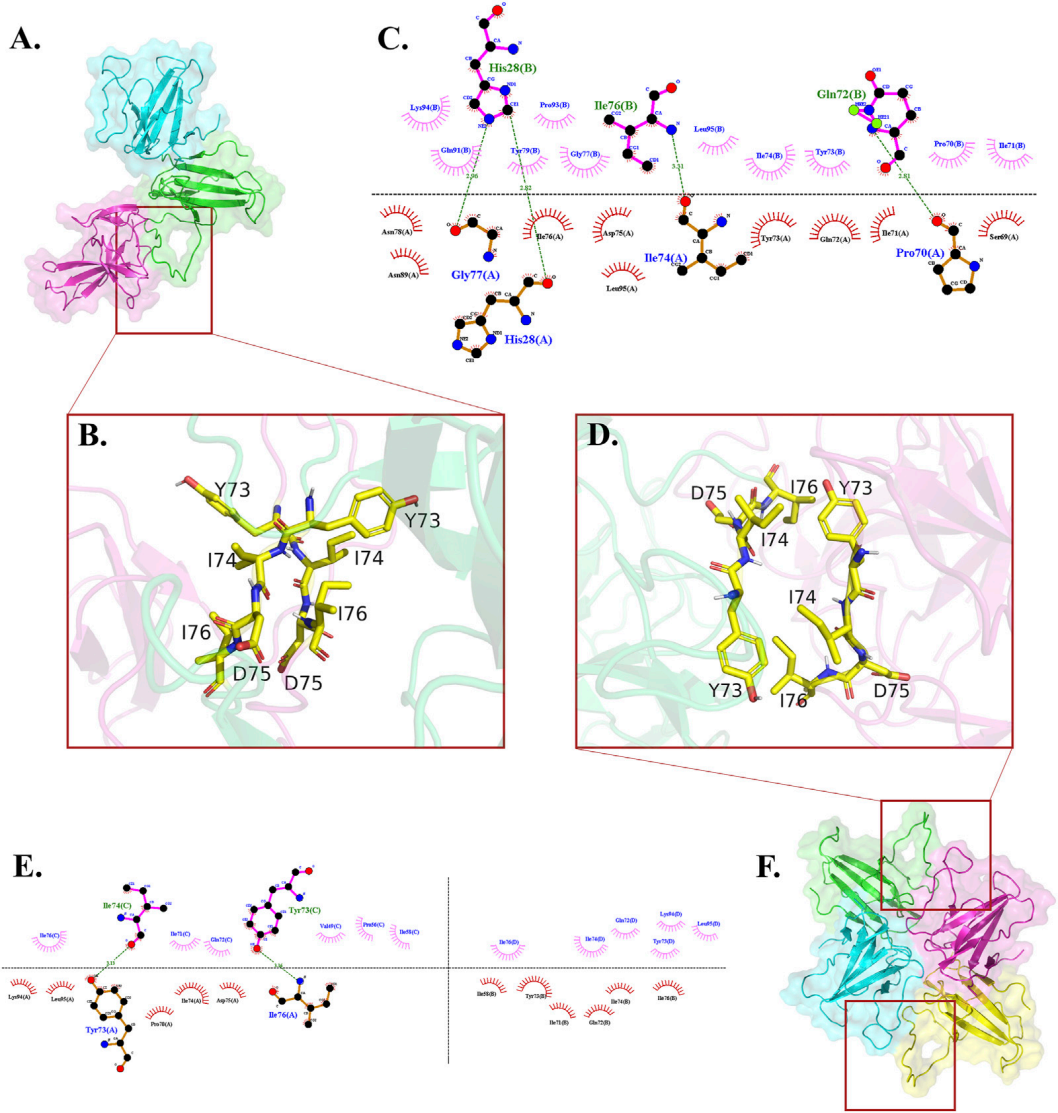
Where the 73YIDI76 motifs could be assembled differently among trimers and tetramers, the interface residues interact hydrophobically with each other. **(A)** Top-ranked trimer structure. **(B)** The 73YIDI76 motifs are shown as sticks are parallel to one another in the trimers. **(C)** The atomistic interactions of the trimer interface residues. **(D)** In the most stable tetramers, the 73YIDI76 motifs are positioned in antiparallel strands next to one another. In this illustration, one of the two dimer linking sites is shown. **(E)** The diagram illustrates the atomistic interactions within the non-covalent interfaces of the tetramer. **(F)** Top-ranked tetramer structure.

In each cluster, merely one chain of dimers, wherein active residues were selected, partaken in the generated interface between the dimer and the monomer (Figures 1A, 2A). The interface residues were approximately equal on the two sides of interface which were assembled identically in the chains. They designed an almost symmetrical pattern in the interface via the parallel orientation of 73YIDI76 motif strands (Figure 2B). In all clusters, Asn89, Lue95, and His28 side chains are engaged in the assembly of the interface by covering the tyrosine side of 73YIDI76 motif in their respective monomer, in contrast to Cys90 and Glu92 side chains, which were never engaged because of their opposite orientation away from the 73YIDI76 motif.

The hydrophobic interactions have a more remarkable contribution to the maintenance of the interface assembly than the polar contacts. Several hydrogen bonds are involved in stabilizing this non-covalent interface, including His28 (A) - His28 (B), Gly77 (A) - His28 (B), Ile74 (A) - Ile76 (B), Pro70 (A) - Gln72 (B) (Figure 2C).

### 3.2 Tetramer structure modeling

In the first set of tetramer models in which 73YIDI76 residues were defined as active residues in one chain of each dimer, 11 clusters containing 122 structures were identified. These tetramer structures have interface RMSD values ranging from 5 to 15, whereas trimer structures typically have values between 0 and 1. Out of the resulting clusters, cluster 11 stands out as the most reliable. In this structurally distinct cluster, all four β4-β5 loops from different monomers contribute to the formation of the tetramer. Alternatively, in other clusters, only two monomers directly form the non-covalent interface among the dimers (Figure 1B).

Interface electrostatic energy of resulting structures ranged from 0 to −80 kcal/mol. Furthermore, Cluster 11 and 4 had Interfaces with van der Waals energy of −40 to −60 kcal/mol, while other clusters were in a range of −15 to −40 kcal/mol (Table 2).

**TABLE 2:** Statistics of tetramers produced by a single defined non-covalent interface. Information of clusters is ordered in the table columns from left to right according to their HADDOCK scores.

Cluster number	11	4	1	2	10	6	7	9	8	3
HADDOCK score	85.3 ± 5.5	−76.7 ± 6.5	−53.8 ± 0.9	−52.1 ± 4.8	−48.0 ± 6.5	−47.9 ± 7.9	−46.6 ± 7.7	−37.9 ± 4.8	−37.3 ± 6.6	−33.5 ± 4.1
Cluster size	4	7	66	10	4	5	5	4	4	8
RMSD from the overall lowest energy structure	17.7 ± 0.3	6.5 ± 0.4	10.7 ± 0.4	6.9 ± 1.2	16.7 ± 0.6	12.4 ± 0.7	11.0 ± 0.7	9.2 ± 1.1	8.6 ± 0.5	12.5 ± 0.8
Van der Waals energy	−54.6 ± 5.8	−53.0 ± 8.4	−32.6 ± 2.8	−33.8 ± 5.5	−29.5 ± 3.7	−33.8 ± 6.2	−30.6 ± 4.9	−22.0 ± 6.1	−22.7 ± 6.1	−21.6 ± 4.4
Electrostatic energy	−43.7 ± 6.4	−50.2 ± 16.7	−57.3 ± 25.4	−57.3 ± 13.5	−60.0 ± 17.5	−13.4 ± 6.7	−44.5 ± 30.9	−44.8 ± 18.9	−6.3 ± 6.0	−19.1 ± 4.7
Desolvation energy	−23.1 ± 1.7	−14.4 ± 0.7	−10.6 ± 1.9	−7.6 ± 1.4	−8.8 ± 0.9	−13.6 ± 1.6	−9.7 ± 2.0	−9.2 ± 1.0	−14.4 ± 1.9	−10.2 ± 1.6
Restraints violation energy	11.0 ± 16.2	8.3 ± 2.5	9.0 ± 13.4	6.8 ± 4.1	23.0 ± 14.7	21.1 ± 13.2	25.6 ± 14.2	22.9 ± 15.6	11.2 ± 4.0	20.9 ± 15.9
Buried Surface Area	1918.8 ± 131.5	1602.3 ± 79.7	1001.5 ± 66.3	1220.8 ± 149.2	1183.9 ± 132.8	1063.1 ± 37.6	975.5 ± 196.3	955.3 ± 166.1	937.5 ± 136.3	777.7 ± 99.1
Z-Score	−2.1	−1.6	−0.1	−0.0	0.2	0.2	0.3	0.9	0.9	1.2

Similar to trimers, the 73YIDI76 motif is situated in the center of the interface, and the amino acids around it contribute to the interaction between dimers. Across the clusters, 73YIDI76 motifs are angled differently from each other. The 73YIDI76 motifs are arranged parallel to one another in clusters 1 to 7 but antiparallel in cluster 11. The 73YIDI76s were arranged head-to-tail in clusters 9 and 10. Representative interfaces of each cluster were visualized comprehensively in the Supplementary Materials.

Each monomer in the tetramers of cluster 11 interacts with the other monomers on two distinct sides: a covalent interface on one side and a non-covalent interface that forms during docking on the other side to produce a shape like a doughnut. There is no major difference between two generated non-covalent interfaces in terms of their structure and interface residues. The existence of two interfaces connecting the dimers in cluster 11 may explain the more extended BSA, and consequently, more interface van der Waals energy compared to the other clusters.

To find the best tetramer conformation that all of YIDI motifs are involved and evaluate the doughnut-like arrangement the second docking set of tetramer was performed. In the second set, 73YIDI76 residues were defined as active residues in both monomers of each dimer according to cluster 11 of the first set. The resulting structures include 173 doughnut-like structures that are grouped into six clusters. The majority of RMSD values for generated structures range between 0 and 6. Cluster three structures have smaller RMSD values than those of other clusters. The van der Waals and electrostatic energies of generated structures ranged between −20 and −95 and 0 and -150, respectively. The structures of cluster 3 have more measurable negative values of interaction energies than the other created tetramers. Moreover, cluster 3 has the most extended BSA of 2946.9 ± 145.7 Å^2^ (Table 3).

**TABLE 3:** Statistics of tetramers constructed by two non-covalent interfaces defined docking.

Cluster number	3	2	4	5	1	6
HADDOCK score	−135.4 ± 4.2	−124.9 ± 4.3	−87.5 ± 4.3	−75.3 ± 14.1	−71.3 ± 1.5	−35.3 ± 5.5
Cluster size	37	38	10	5	79	4
RMSD from the overall lowest energy structure	1.0 ± 0.6	2.5 ± 0.2	6.1 ± 0.3	1.4 ± 0.2	3.6 ± 0.2	6.3 ± 0.3
Van der Waals energy	−85.7 ± 6.6	−80.6 ± 4.2	−50.0 ± 4.3	−48.0 ± 6.8	−43.2 ± 2.1	−24.0 ± 2.7
Electrostatic energy	−105.4 ± 30.1	−83.3 ± 7.2	−51.2 ± 4.5	−39.3 ± 25.5	−13.5 ± 4.5	0.2 ± 3.6
Desolvation energy	−32.8 ± 1.5	−33.0 ± 2.2	−33.8 ± 1.6	−25.2 ± 3.1	−27.6 ± 2.5	−19.0 ± 0.4
Restraints violation energy	42.0 ± 24.5	53.2 ± 23.5	65.4 ± 20.3	57.8 ± 14.8	22.2 ± 15.8	76.5 ± 25.2
Buried Surface Area	2946.9 ± 145.7	2532.9 ± 108.6	1944.9 ± 94.5	2018.1 ± 243.8	1739.3 ± 102.5	1082.6 ± 71.0
Z-Score	−1.4	−1.1	0.0	0.4	0.5	1.6

Structures of different clusters show a similar arrangement pattern. All four monomers participate in tetramer formation by constructing two non-covalent interfaces between the dimers (Figure 1C).

The center of each interface is formed with 73YIDI76 residues that are arranged antiparallel to one another (Figure 2D). 73YIDI76’s orientation angles, however, slightly vary among the clusters. Residues engaged in the interface, except 73YIDI76 residues, change across various clusters. However, only a small number of residues, such as leucine 95, are conserved across all interfaces. Like the trimers, the hydrophobic interactions primarily support the interface’s structural assembly. The Tyr73 side chain can form hydrogen bonds via the backbone of opposite chain residues such as Ile74 or Ile76 (Figure 2E).

The top-ranked doughnut-like tetramer was selected to perform the molecular dynamics simulations and evaluate the construct stability (Figure 2F).

### 3.3 Global dynamics of ORF8 trimer and tetramer

The overall RMSD plots show that the doughnut-like tetramers are considerably more stable than the trimeric forms due to their RMSD range, and changes. The average RMSD values of tetrameric and trimeric ORF8 are 4 and 5.9 Å, respectively.

**FIGURE 3:**
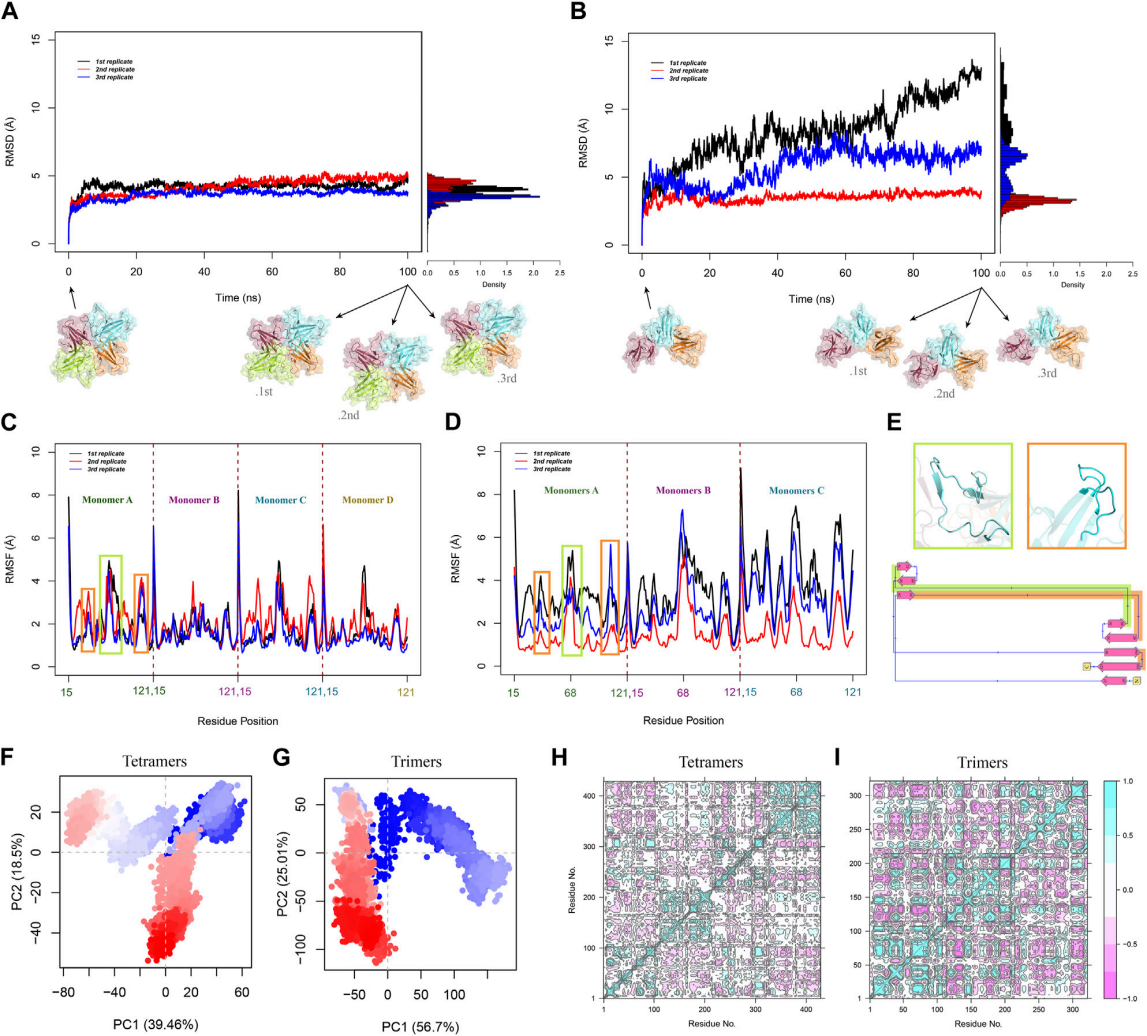
Overall RMSD of Cα atoms in **(A)** tetramers and **(B)** trimers was calculated over 100 ns for all replications. RMSD time-dependent plots show the RMSD changes in the system and the histograms demonstrate the RMSD distribution. RMSF analysis of Cα atoms was performed for all the MD replications of **(C)** tetramers and **(D)** trimers. **(E)** The green box displays cartoon representation of the β4-β5 loop and the orange box represents the MHC-1 direct binding site. These flexible regions are highlighted with the corresponding colors on the 2D diagram of the monomer. PCA plots illustrated the distribution and clustering of **(F)** tetrameric and **(G)** trimeric ORF8 conformations in the PC space. Each point represents a frame from the trajectories, and the shades of red and blue represent the progression of time. The residue cross-correlation matrices of **(H)** tetramers and **(I)** trimers. pink and cyan spots present the atom’s correlated and anti-correlated motions, respectively.

In the tetrameric form, the RMSD reaches a relatively stable plateau after 10 (Figure 3A). This indicates that the tetrameric form attains a relatively stable conformation with minor structural deviations throughout the simulation.

However, compared to the tetrameric form, the RMSD plots of the trimeric form show more variations. Trimers exhibit more significant conformational changes and a larger degree of flexibility, as seen by the observed diversity in RMSD changes throughout the three sets of simulations, Inferring the overall lower stability of the trimeric forms compared to the tetramers (Figure 3B). RMSD histograms exhibit a narrow distribution (from 0 to 5 Å) with a peak around 4 Å for tetrameric form. In contrast, the trimeric form shows a broader distribution from 0 to 15 Å, reflecting a greater conformational variability within the ensemble.

To study the flexibility and local fluctuations within the trimeric and tetrameric ORF8, RMSF analysis was performed. Average RMSF values for tetrameric and trimeric ORF8 are 1.8 Å and 2.6 Å, respectively. The tetrameric form exhibits overall lower RMSF values, suggesting a more rigid structure compared to the more flexible trimeric form (Figures 3C, D).

Most residues in the tetrameric and trimeric ORF8 have relatively low fluctuations in their RMSF profile; however, because of their structural flexibility, residues 47-83 (the ORF8 interacting loop between β4 and β5) and 104-110 (the proposed MHC-I binding site) have higher RMSF values (Figure 3E). A similar RMSF profile and the considerably higher flexibility of β4-β5 loop region were previously observed in MD studies on ORF8 dimer structures (Chaudhari et al., 2023; Islam et al., 2023; Selvaraj et al., 2023). These flexible regions were suggested as functional regions that may play major roles in protein-protein interactions in past studies (Wang et al., 2021; Cheng and Peng, 2022; Arduini et al., 2023). These regions may undergo conformational changes during their functional processes. The average RMSF of β4-β5 loops of the tetramers is relatively lower in comparison to that of trimers indicating that the loops are more flexible in trimers. This suggests that the more extended non-covalent interface area among the dimers in the tetrameric form restrains the loop fluctuations.

PCA was performed on the MD trajectories to gain insights into the conformational dynamics and structural differences between ORF8 trimer and tetramer.

The percentages of variance captured by PC1 and PC2 of the systems are tetramer 39.46% and 18.5%, respectively, and trimer 56.7% and 25.01%. The first four principal components of the tetramer, and the first two principal components of the trimer capture more than 70% of the total variance.

Since all of the monomers are directly involved during tetramerization, the system’s fluctuations are dampened, as seen by the tetrameric system’s lower variance when compared to the trimer. PCA projection plot of the tetrameric system reveals dense clusters of conformations around the initial point, indicating that the complex explores a relatively narrow conformational space (Figure 3F). The plot of the trimeric system displays a more dispersed distribution of conformations, suggesting more structural variety (Figure 3G). This shows that the tetrameric assembly has a narrower range of motion, and less structural diversity than the trimeric ORF8.

DCCM analysis was performed to study the correlated and anti-correlated motions within the oligomeric forms of ORF8.

In the tetrameric form, the DCCM displays a reasonably preserved pattern of prolonged coupled movements inside the chains with strong correlation coefficients. The DCCM map of tetrameric constructions illustrates the extensive anticorrelated movements of the monomers that are next to one other (A-B, B-C, and C-D). While the monomers that are not directly connected (A-C and B-D) show a different pattern, they have correlated motions in some regions that may be affected by the tetramer systemic conformational changes (Figure 3H). The time-dependent changes in distance between the center of mass of these monomers (A-C and B-D) show that the COM of monomers A and C were approaching each other during the simulation. In contrast, the COM of monomers B and D move away from each other (Supplementary Figure S12).

Similarly, the DCCM of trimeric constructs highlights a conserved pattern for interchain motions but indicates different patterns for the intrachain motions (Figure 3I).

### 3.4 The interacting β4-β5 loop dynamics throughout the oligomerization of ORF8

In the tetramer subunit-wise RMSD analysis, each monomer exhibits a similar pattern of RMSD changes (Supplementary Figure S13A), while the subunit B of trimers has a distinct pattern, indicating variations in structural fluctuations that may be caused by interchain interactions via non-covalent interface. In contrast, the subunits A and C show a relatively low and stable RMSD (Supplementary Figure S13B).

The MD trajectories of the oligomeric forms of ORF8 underwent secondary structure analysis, which provided insights into the dynamics of disordered loops, such as the extended β4-β5 loop, which is essential for the formation of the non-covalent interface, and the conformational stability of the extended configurations. The tetrameric and trimeric forms maintained a well-defined secondary structure in the ß-strands regions of the protein, consistent with the known structural features of ORF8 (Supplementary Figures S14, S15).

In all replicates of tetrameric and trimeric forms, β4-β5 loop displayed a predominantly random coil conformation, with occasional transitions to a 3_10_ helix at position 47-50. The middle of the loop interruptedly folds into 3_10_ helices throughout some trajectories.

The secondary structure and RMSF analysis highlight the dynamic nature of the β4-β5 loop and its potential role in mediating protein-protein interactions in the ORF8 tetramer and trimer.

### 3.5 MHC-I binding site, covalent interface, DGBM, and β4-β5 loop dynamics in the oligomeric constructs

**FIGURE 4:**
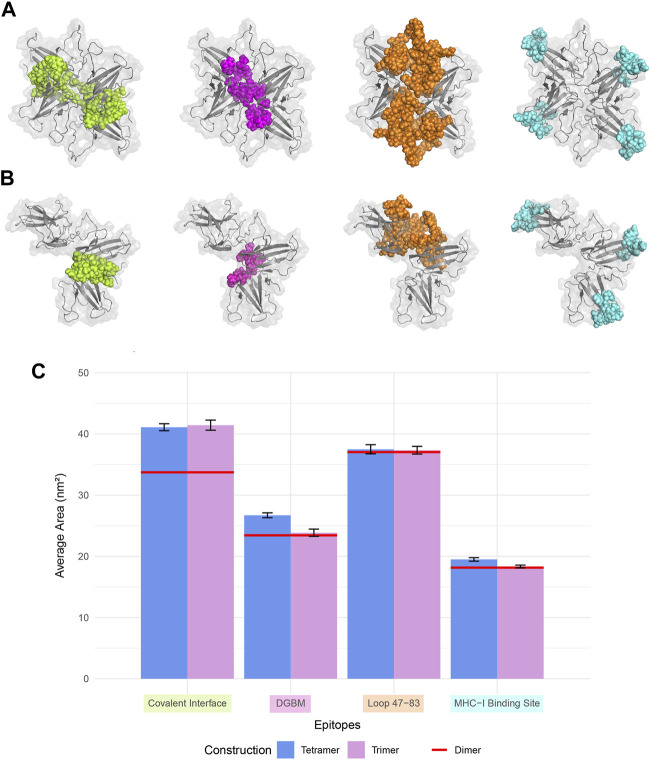
Four regions that are crucial for the protein-protein interactions, and drug design were considered for SASA analysis. The covalent interface (lime), DGBM (light magenta), residues 47-83 (tv_orange), and MHC-I binding site (cyan) are represented in sphere style on ORF8 tetramer and trimer in **(A)** and **(B)**, respectively. **(C)** The SASA was calculated over 100 ns MD simulation for trimers and tetramers, and compared to that of the dimer. There were no statistically significant differences among the constructions in the regions' SASA values except for the covalent interface and DGBM. The tetrameric forms exhibit slightly more solvent accessibility for the MHC-I direct binding site.

Based on different binding poses and interfaces suggested in previous studies and drug designs based on ORF8 structure and interactions, four specific regions are considered important hotspots: the dimerization covalent interface, DGBM, the β4-β5 loop, and the MHC-I direct binding site (Figures 4A, B).

**FIGURE 5:**
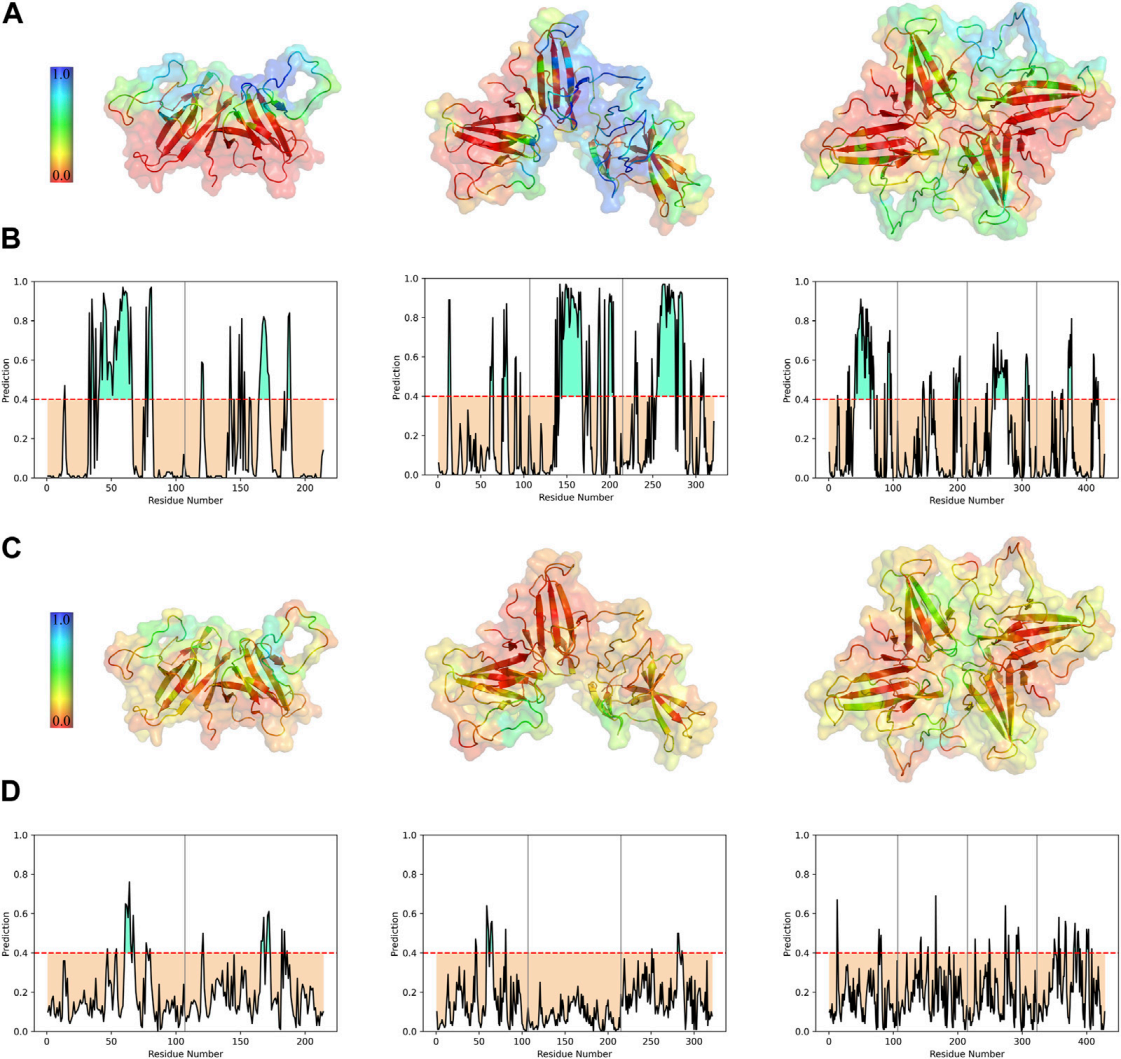
Interface prediction of different ORF8 constructs. Predicted protein-protein **(A)** and protein-ligand **(C)** binding interfaces of different constructs are colored with a spectrum of colors based on the interaction probability. The per-residue prediction results of protein-protein **(B)** and protein-ligand **(D)** interfaces were represented as plots. Chains are separated by vertical gray lines on the plots.

The analysis of protein-protein binding sites using PeSTo protein structure transformer indicated that, in tetramers, β4-β5 loop experiences a reduction in its overall availability for interactions with other proteins. On the other hand, in trimers, these loops provide a fascinating possibility since they include residues that have a high likelihood of interacting. The saddle-shaped structure that develops at the non-covalent interface in trimers may be related to this phenomenon. It remains to be determined if this saddle-shaped region performs any function. Tetramers and trimers exhibit higher binding potentials than dimers at the MHC-I direct binding site. (Figures 5A, B).

Studying the potential binding surfaces for small molecules indicated that tetramers and trimers have a slightly more potential residue than dimers. This suggests that the solvent and ligand molecules can still bind to the central parts of tetrameric ORF8, including the covalent interface and DGBM, despite restricted access of proteins to these regions. Furthermore, the results of solvent accessibility analysis, and structural visualization point to the possibility of a channel-like area in the center of the tetramer. The central region of β4-β5 loop in dimers showed a higher binding potential for ligands, which decreased in trimers (especially in the chain involved in oligomerization), and reached its lowest value in the tetramer, indicating that oligomerization could affect the binding potential of this region through interchain interactions (Figures 5C, D). On the other hand, concerning the SASA values of the β4-β5 loop, there are no statistically significant differences between the three different assemblies (Figure 4C). However, in tetrameric and trimeric forms, the covalent interface and DGBM continue to be promising targets for drug design.

Epitope predictions using the ElliPro server, which are based on structure protrusion, suggest relatively conserved surfaces in the dimer, trimer, and tetramer forms of ORF8 (Supplementary Figure S16). The absence of protrusion of the β4-β5 loops inside the non-covalent interface of the trimeric structure results in the loss of their capacity to serve as B-cell epitopes. While, in terms of the tetrameric non-covalent interfaces being oriented differently, the borders of β4-β5 loops are exposed and could introduce new B-cell epitopes. Moreover, the predicted epitopes include the direct binding region to MHC-I that is capable of acting as a B-cell epitope allowing ORF8 to induce an immune responce.

## 4 Discussion

Due to ORF8 uninvestigated high-order assemblies, its interactions, and functions are not entirely understood. On the other hand, ORF8 could be a profitable candidate with high druggability for developing new COVID-19 therapeutics (Valcarcel et al., 2021). To make advancements in this issue, molecular modeling tools were used to construct and study the dynamics of trimeric and tetrameric ORF8 structures, and the potential binding sites were studied in the oligomeric forms.

The results show that stable ORF8 tetramers and trimers may be formed when β4-β5 loops are close to one another. The trimers' 73YIDI76 motif parallel orientations matched the crystallographic contact discovered in X-ray crystallography studies (Flower et al., 2021). As for the positioning angle of 73YIDI76 motifs next to each other, tetramers showed a wide range of variation. The most stable arrangement for the tetramers is the form in which four β4-β5 loops are assembled as two identical interfaces among the dimers. Despite the trimers and crystallographic contact, in these doughnut-like tetramers, the 73YIDI76 motifs are antiparallel to one another.

To study the dynamical behavior and stability of oligomeric forms of ORF8 MD simulations were performed for each system. The analyses show that the doughnut-like tetramers exhibit noticeably higher stability compared to the trimeric forms. As evidenced by their smaller RMSD values over the simulations and more constrained conformational space in the PCA analysis.

On the other hand, trimer models manifest significant changes in the RMSD plots of two replicates simulations.

The higher stability of the tetramers and the trimer’s relatively lower stability supports the previous experimental results that separate dimers and tetramers as functional forms of this protein (Kumar et al., 2023).

By comparing the average RMSD values for the trimeric system (5.9 Å), and the tetrameric system (4 Å) with the values previously reported for the dimeric form, it offers insight into the varying degrees of stability among these systems. The average RMSD for the native ORF8 dimer was reported to be 2.4 Å over 1 μs of the simulation time (Islam et al., 2023).

As expected, β4-β5 loop region and the MHC-I binding site demonstrate higher RMSF values. In addition, the examination of the secondary structure further emphasizes the inherent flexibility of the β4-β5 loop, which serves as a critical mediator of interactions in both the tetrameric and trimeric configurations of ORF8. It has been observed that the manipulation of disulfide bonds can impact the conformation of the β4-β5 loop segments (Cheng and Peng, 2022). Further studies can investigate the ORF8 multimeric forms about the oxidative and reductive environments.

Analyzing correlated, and anti-correlated motions within the oligomeric assemblies using DCCM, revealed that the chains maintain their general motion patterns in different oligomeric forms.

The oligomeric structure of ORF8 can provide a framework for structural studies of ORF8 protein-protein interactions. The 73YIDI76 motifs could be occupied by host proteins, preventing ORF8 oligomers from enduring. On the other hand, oligomer formations can decrease the interaction of the loop with other partners. Furthermore, the ORF8 interactions can be enhanced if the interaction sites, such as the MHC-1 binding site, remain exposed in the multimeric forms. The protrusion and high flexibility of the MHC-I direct binding sites are maintained in oligomeric forms contributing to their immunogenicity, and interaction with their partners. The β4-β5 loops are shown to be engaged in the oligomeric configuration. However, because to their inherent flexibility, these loops have the ability to extend outward in certain conformations, so generating a distinct surface potential that serves as a novel epitope in the tetrameric structure.

**FIGURE 6:**
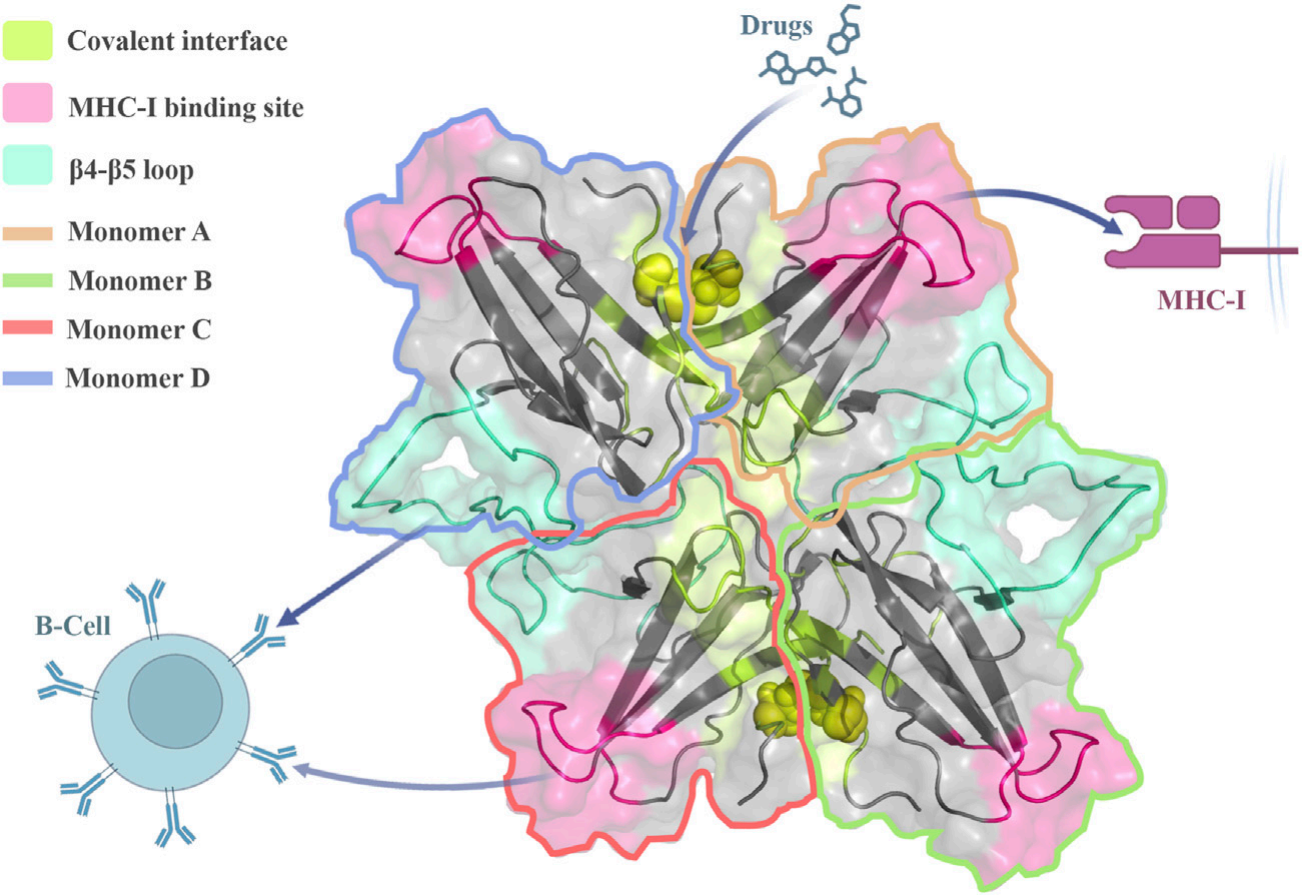
Stable ORF8 oligomeric forms can interact with immune system components, and regulate the immune response. The oligomerization sites in trimeric and tetrameric forms are accessible to drugs proposing potential hotspots for disrupting ORF8 function and assembling.

In terms of binding potential and SASA analyses, the drug target hotspots remained accessible during the oligomerization process. As previously suggested, when specific drugs target the covalent interface, they can disrupt the interchain disulfide bond and dimer formation (Selvaraj et al., 2023). Therefore, the loss of dimerization capability prevents ORF8 from interacting with MHC-I which reduces the immune system evasion (Chaudhari et al., 2023). Consequently, the drugs that target the interacting β4-β5 loop (non-covalent interfaces) or the covalent interfaces could interrupt the oligomerization, and affect the protein pathogenesis activities (Figure 6). Finally, all of these considerations may create insight into how the ORF8 causes immune evasion and lead to the development of new COVID-19 treatments.

Future research initiatives may explore the potential influence of inhibitors, mutations, and glycosylation on the process of ORF8 oligomerization and its associated relationships. Furthermore, future research may delve into the examination of the quantities and localization of different oligomeric forms of ORF8 under diverse physiological situations.

## 5 Conclusion

ORF8 is SARS-CoV-2 accessory protein that maintains the viral replication and pathogenesis. Its structural characteristics, such as Ig-like folding, and the flexible β4-β5 loop containing the 73YIDI76 motif, allow it to interact with the immune system and assemble high-order structures.

Molecular modeling of ORF8 oligomeric constructs indicated that it assembles as stable tetramers via β4-β5 loop. The tetramers, in which all the monomers directly interact with each other and form a doughnut-like assembly of subunits, seem to be the most stable oligomers. Numerous protein interactions involved in immune evasion and inflammatory processes may be impacted by this oligomerization mechanism. Nonetheless, oligomerization has no discernible effect on the MHC-I epitopes, which continue to function normally. Considering solvent accessibility, and binding potential for covalent interface in the oligomeric forms it can be targeted by small molecules to disrupt the oligomerization.

Finally, the oligomeric ORF8 structures could contribute to further structural studies and the development of new antiviral medications.

## Data Availability

The original contributions presented in the study are included in the article/Supplementary Materials, further inquiries can be directed to the corresponding author.
