# Supplementary material for: Oligomer formation of SARS-CoV-2 ORF8 through 73YIDI76 motifs regulates immune response and non-infusion antiviral interactions
Source: Front Mol Biosci. 2023 Nov 29;10:1270511. doi: 10.3389/fmolb.2023.1270511 (PMC10716485; doi:10.3389/fmolb.2023.1270511)
Supplement: Supplementary file 1 [file DataSheet1.PDF]

Supplementary Materials

Oligomer Formation of SARS-CoV-2 ORF8 Through 73YIDI76 Motifs Regulates Immune Response and Non-infusion Antiviral Interactions

Mohammad Assadizadeh [1] and Maryam Azimzadeh Irani [1*]

[1] Faculty of Life Sciences and Biotechnology, Shahid Beheshti University, Tehran, Iran.
Corresponding author email: *m_azimzadeh@sbu.ac.ir

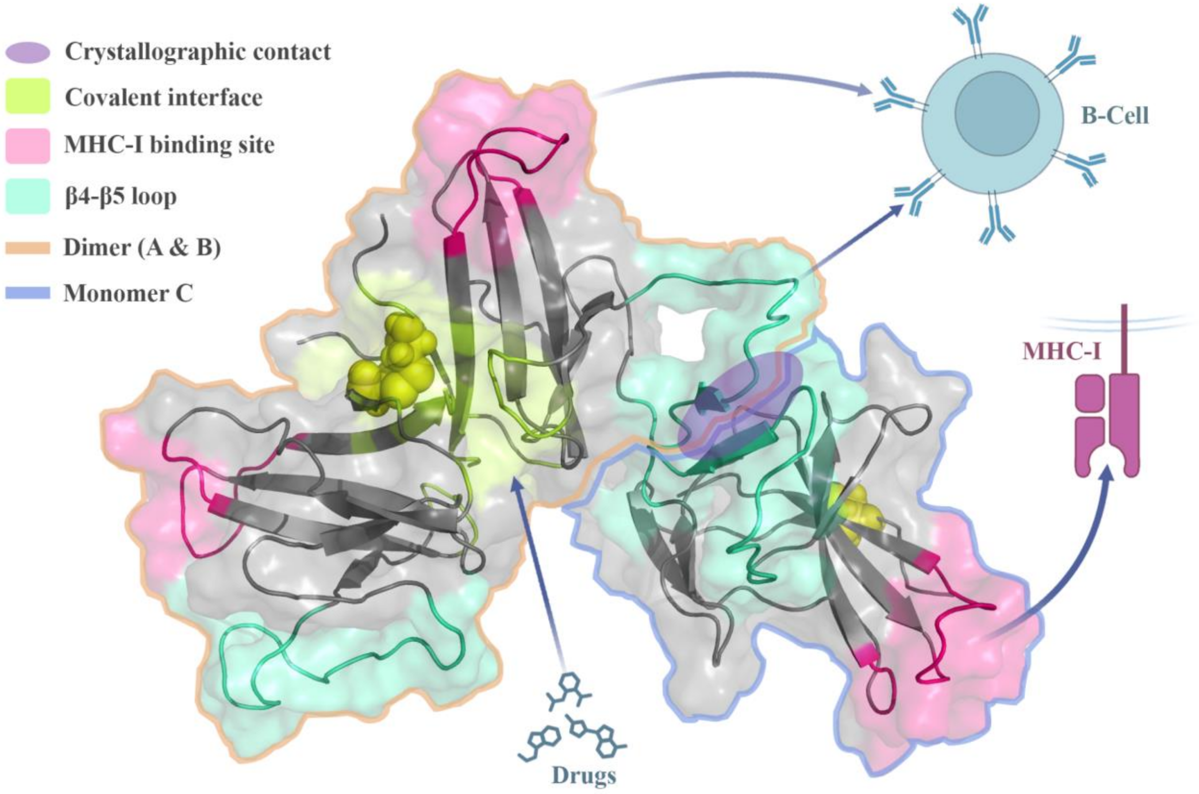

Figure S1. The ORF8 dimer is proposed to be able to interact with other subunits through the crystallographic contact obtained in the crystal lattice.

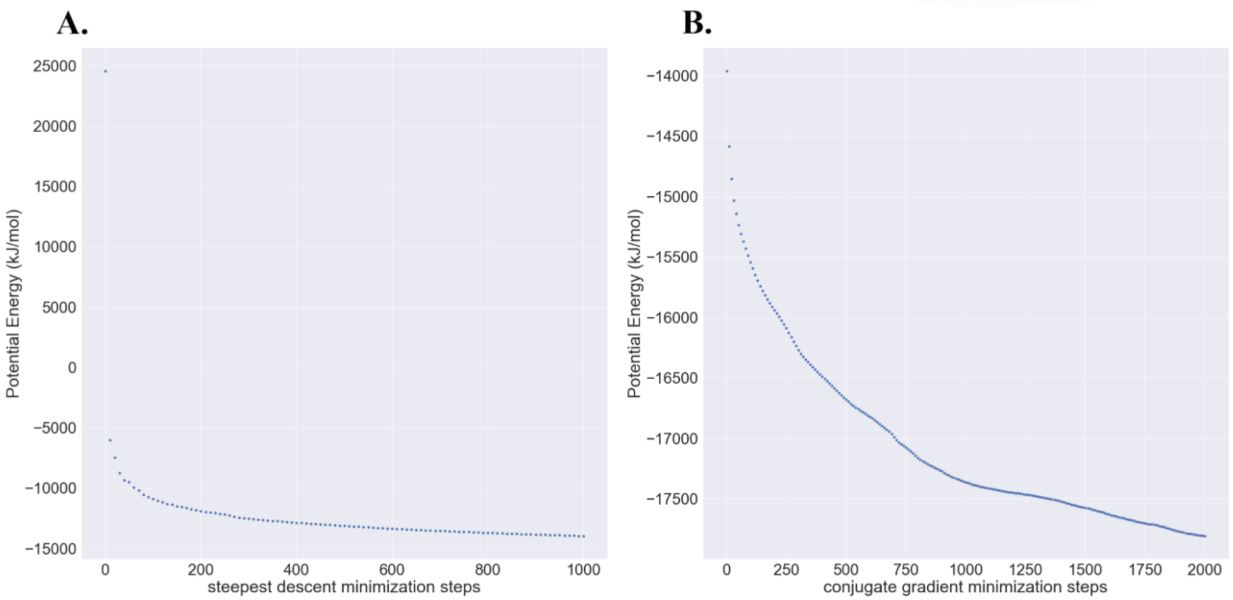

Figure S2. Scatter plot representation of energy minimization process prepared dimer. **(A)** Energy reduction through steepest descent minimization in 1000 steps. **(B)** Energy reduction through conjugate gradient minimization in 2000 steps.

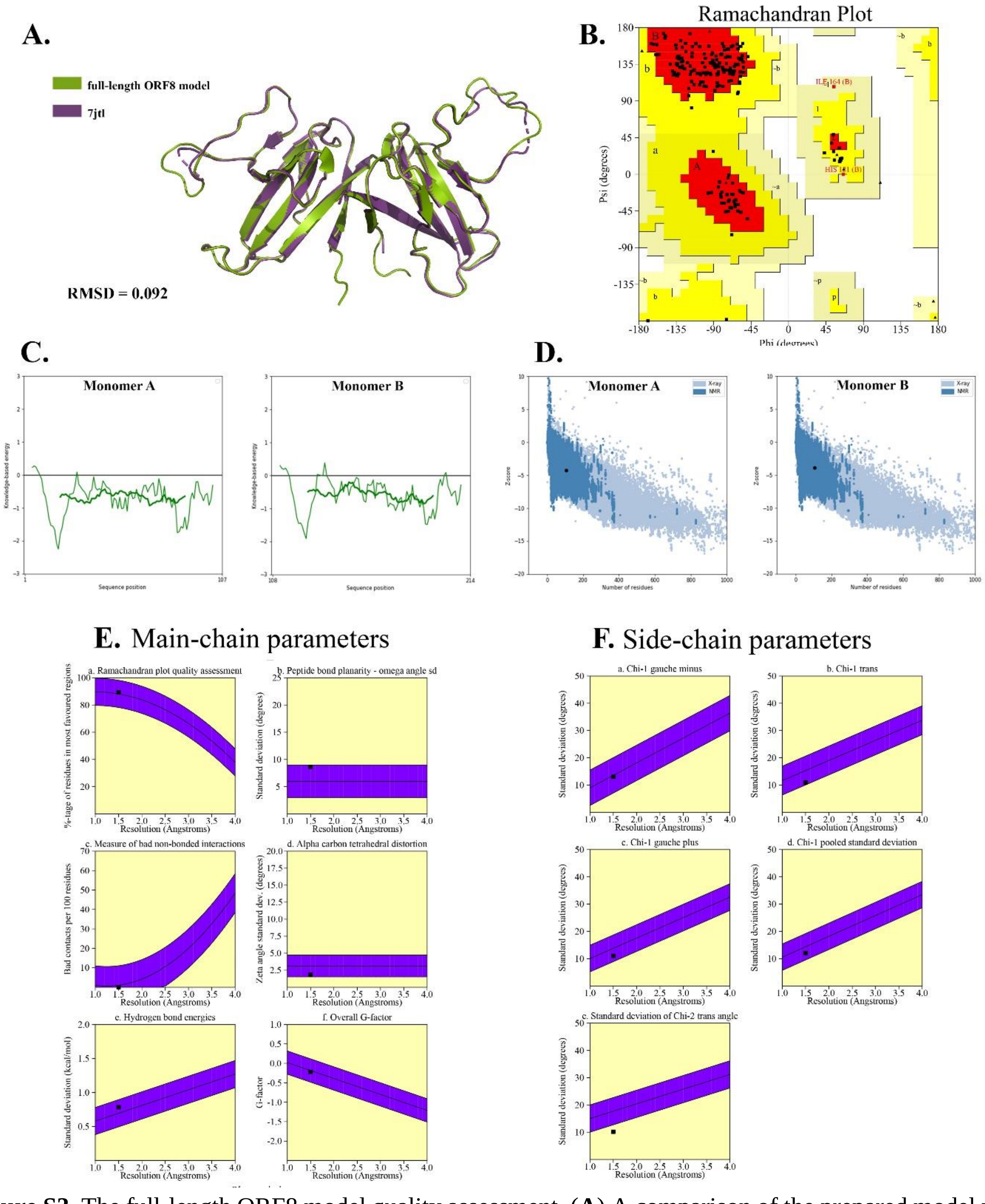

Figure S3. The full-length ORF8 model quality assessment. (**A**) A comparison of the prepared model with the 7JTL model reveals inconsistent conformational changes. (**B**) Based on the Ramachandran plot, it has been observed that none of the residues present are situated in disallowed regions. Further, an overwhelming majority of around 90% of the residues are positioned in the most favored regions. (**C**) The energy plots displayed negative values for each monomer that indicated the appropriate 3D structure. (**D**) The overall model quality can be investigated based on the Z-score plot. The Z-score for the dimer model is located within the range of Z-scores for experimental structures. (**E**) and (**F**) show the standard deviation of stereochemical parameters at different structural resolutions. The dimer model falls within the range of experimental data standard deviation.

Trimer docking

Cluster number	1	2	3
HADDOCK score	-101.6 +/- 2.0	-72.3 +/- 6.4	-56.4 +/- 3.5
Cluster size	170	21	4
RMSD	1.4 +/- 1.0	1.4 +/- 0.4	4.7 +/- 0.4
Van der Waals energy	-71.8 +/- 2.1	-46.5 +/- 5.0	-38.5 +/- 3.9
Electrostatic energy	-62.6 +/- 9.7	-55.0 +/- 5.6	-32.7 +/- 7.6
Desolvation energy	-17.9 +/- 1.7	-16.3 +/- 1.2	-13.1 +/- 1.6
Restraints violation energy	6.3 +/- 3.8	14.2 +/- 6.7	17.4 +/- 14.4
Buried Surface Area	1565.7 +/- 61.3	1186.2 +/- 51.2	1057.4 +/- 90.6
Z-Score	-1.3	0.2	1.1

Table S1. Statistics of trimer docking results.

A. cluster 1_1

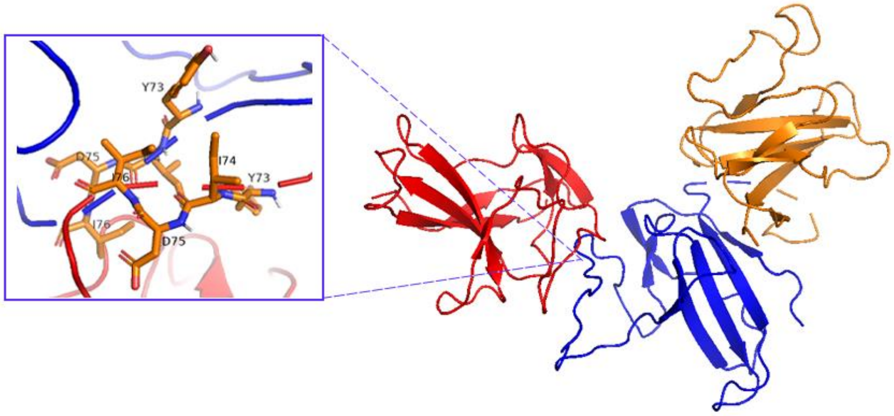


```
15    21   26   31   36   41   46   51   56   61   66   71   76   81   86   91   96   101  106  111  116  121
SNAQECSLQSCTQHQPYVVDDPCPIHFYSKWYIRVGARKSAPLIELCVDEAGSKSPIQYIDIGNYTVSCLPFTINCQEPKLGSLVVRCSFYEDFLEYHDVRVVLDFI
15    21   26   31   36   41   46   51   56   61   66   71   76   81   86   91   96   101  106  111  116  121
SNAQECSLQSCTQHQPYVVDDPCPIHFYSKWYIRVGARKSAPLIELCVDEAGSKSPIQYIDIGNYTVSCLPFTINCQEPKLGSLVVRCSFYEDFLEYHDVRVVLDFI
```

B. cluster 2_1

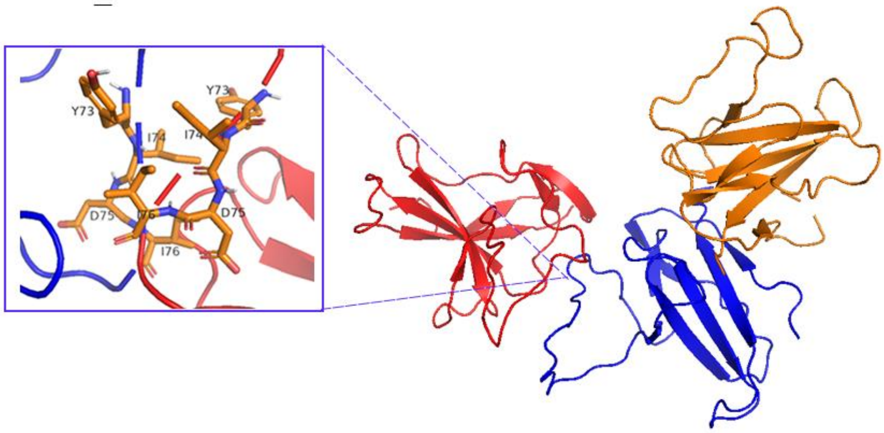


```
15    21   26   31   36   41   46   51   56   61   66   71   76   81   86   91   96   101  106  111  116  121
SNAQECSLQSCTQHQPYVVDDPCPIHFYSKWYIRVGARKSAPLIELCVDEAGSKSPIQYIDIGNYTVSCLPFTINCQEPKLGSLVVRCSFYEDFLEYHDVRVVLDFI
15    21   26   31   36   41   46   51   56   61   66   71   76   81   86   91   96   101  106  111  116  121
SNAQECSLQSCTQHQPYVVDDPCPIHFYSKWYIRVGARKSAPLIELCVDEAGSKSPIQYIDIGNYTVSCLPFTINCQEPKLGSLVVRCSFYEDFLEYHDVRVVLDFI
```

C. cluster 3_1

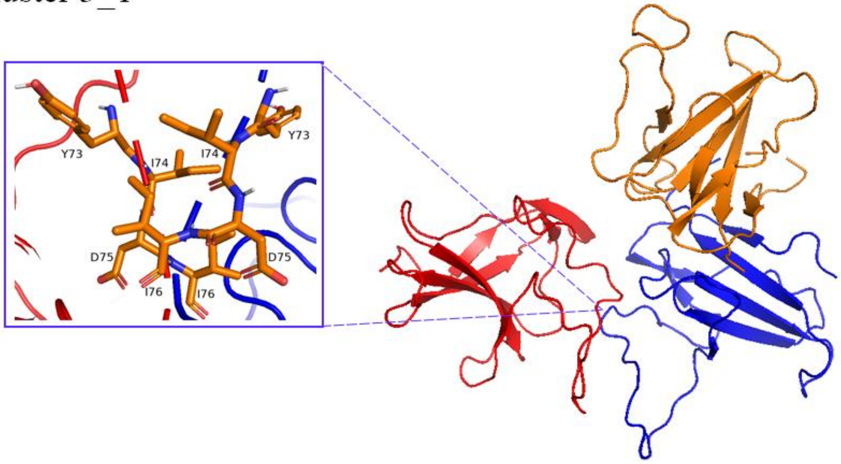


```
15    21   26   31   36   41   46   51   56   61   66   71   76   81   86   91   96   101  106  111  116  121
SNAQECSLQSCTQHQPYVVDDPCPIHFYSKWYIRVGARKSAPLIELCVDEAGSKSPIQYIDIGNYTVSCLPFTINCQEPKLGSLVVRCSFYEDFLEYHDVRVVLDFI
15    21   26   31   36   41   46   51   56   61   66   71   76   81   86   91   96   101  106  111  116  121
SNAQECSLQSCTQHQPYVVDDPCPIHFYSKWYIRVGARKSAPLIELCVDEAGSKSPIQYIDIGNYTVSCLPFTINCQEPKLGSLVVRCSFYEDFLEYHDVRVVLDFI
```

Tetramer docking

Set 1

Cluster number	11	4	1	2	10	6	7	9	8	3
HADDOCK score	-85.3 +/- 5.5	-76.7 +/- 6.5	-53.8 +/- 0.9	-52.1 +/- 4.8	-48.0 +/- 6.5	-47.9 +/- 7.9	-46.6 +/- 7.7	-37.9 +/- 4.8	-37.3 +/- 6.6	-33.5 +/- 4.1
Cluster size	4	7	66	10	4	5	5	4	4	8
RMSD	17.7 +/- 0.3	6.5 +/- 0.4	10.7 +/- 0.4	6.9 +/- 1.2	16.7 +/- 0.6	12.4 +/- 0.7	11.0 +/- 0.7	9.2 +/- 1.1	8.6 +/- 0.5	12.5 +/- 0.8
Van der Waals energy	-54.6 +/- 5.8	-53.0 +/- 8.4	-32.6 +/- 2.8	-33.8 +/- 5.5	-29.5 +/- 3.7	-33.8 +/- 6.2	-30.6 +/- 4.9	-22.0 +/- 6.1	-22.7 +/- 6.1	-21.6 +/- 4.4
Electrostatic energy	-43.7 +/- 6.4	-50.2 +/- 16.7	-57.3 +/- 25.4	-57.3 +/- 13.5	-60.0 +/- 17.5	-13.4 +/- 6.7	-44.5 +/- 30.9	-44.8 +/- 18.9	-6.3 +/- 6.0	-19.1 +/- 4.7
Desolvation energy	-23.1 +/- 1.7	-14.4 +/- 0.7	-10.6 +/- 1.9	-7.6 +/- 1.4	-8.8 +/- 0.9	-13.6 +/- 1.6	-9.7 +/- 2.0	-9.2 +/- 1.0	-14.4 +/- 1.9	-10.2 +/- 1.6
Restraints violation energy	11.0 +/- 16.2	8.3 +/- 2.5	9.0 +/- 13.4	6.8 +/- 4.1	23.0 +/- 14.7	21.1 +/- 13.2	25.6 +/- 14.2	22.9 +/- 15.6	11.2 +/- 4.0	20.9 +/- 15.9
Buried Surface Area	1918.8 +/- 131.5	1602.3 +/- 79.7	1001.5 +/- 66.3	1220.8 +/- 149.2	1183.9 +/- 132.8	1063.1 +/- 37.6	975.5 +/- 196.3	955.3 +/- 166.1	937.5 +/- 136.3	777.7 +/- 99.1
Z-Score	-2.1	-1.6	-0.1	-0.0	0.2	0.2	0.3	0.9	0.9	1.2

Table S2. Statistics of tetramers produced by a single defined non-covalent interface. Information of clusters is ordered in the table columns from left to right according to their HADDOCK scores.

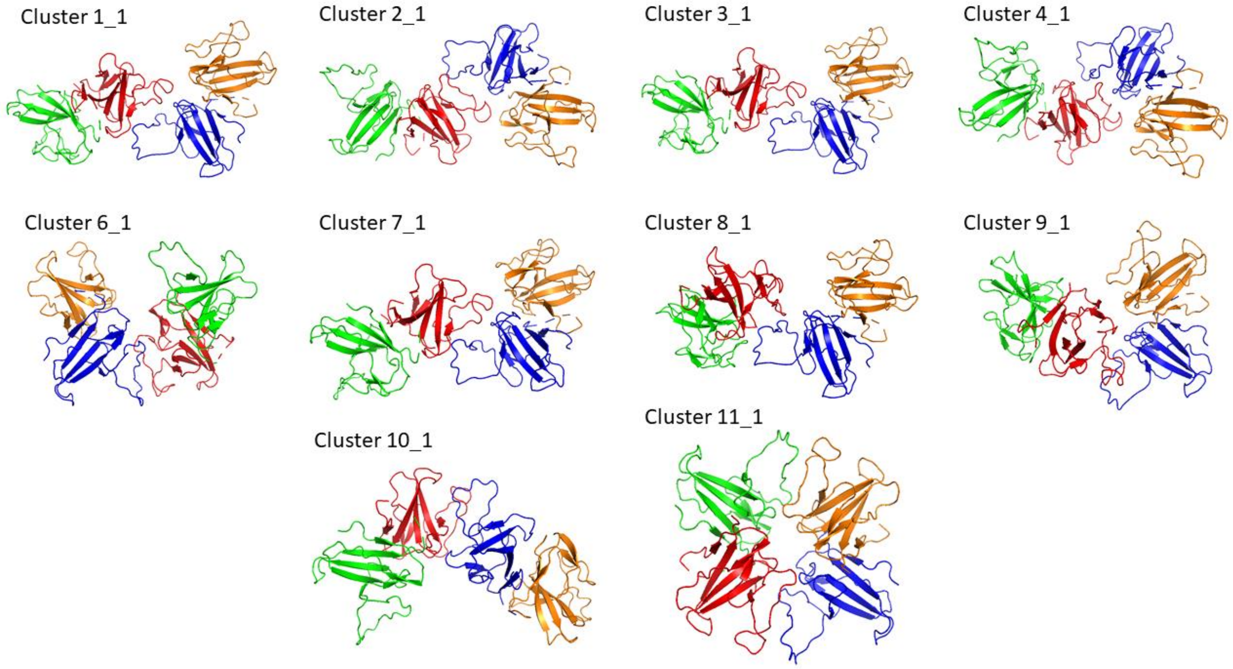

Figure S4. Cartoon representations of all the clusters' best structures from the docking for which a single interface was defined.

A. Cluster 1_1

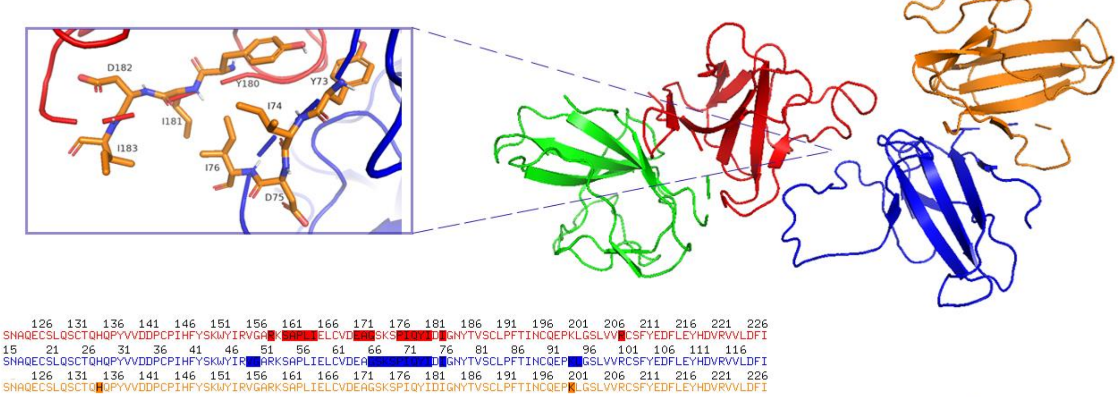

B. Cluster 2_1

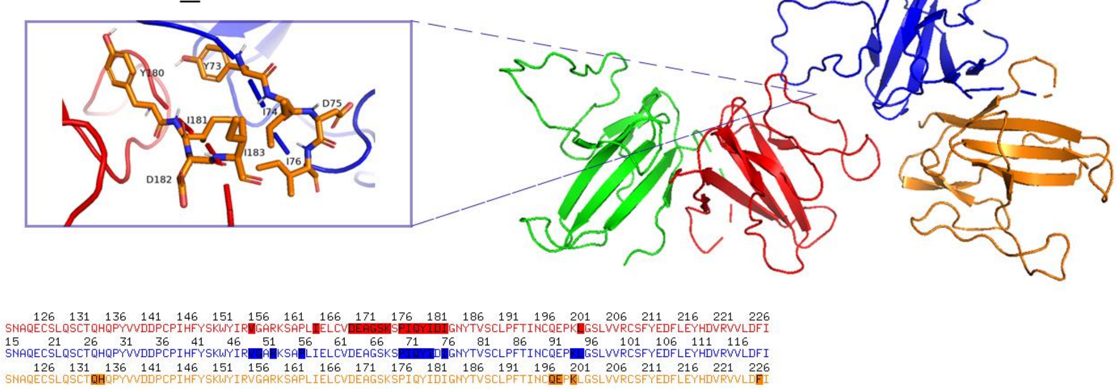

C. Cluster 3_1

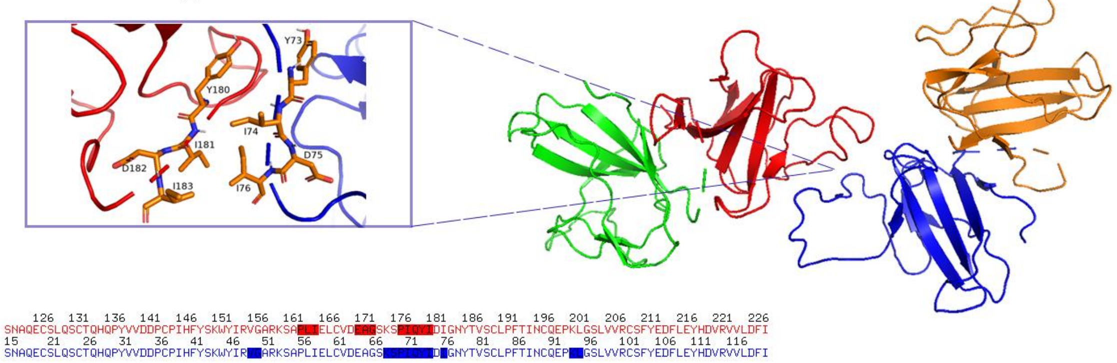

D. Cluster 4_1

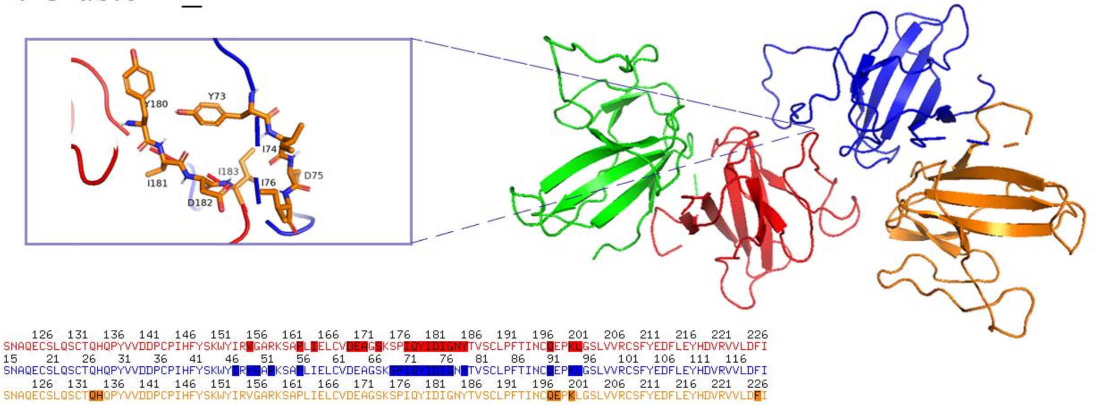

A. Cluster 6_1

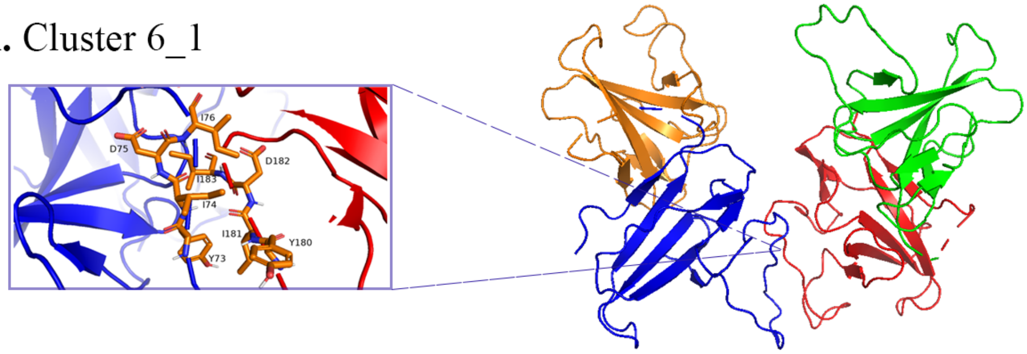


```
   126  131  136  141  146  151  156  161  166  171  176  181  186  191  196  201  206  211  216  221  226
SNAQECSLQSCTQHQPYVVDDPCPIHFYSKWYIRVGARKSAPLIELCVDEAGSKSPIQYIDIGNYTVSCLPFTINCQEPKLGSLVVRCSFYEDFLEYHDVRVVLDFI
15    21   26   31   36   41   46   51   56   61   66   71   76   81   86   91   96   101  106  111  116
SNAQECSLQSCTQHQPYVVDDPCPIHFYSKWYIRVGARKSAPLIELCVDEAGSKSPIQYIDIGNYTVSCLPFTINCQEPKLGSLVVRCSFYEDFLEYHDVRVVLDFI
```

B. Cluster 7_1

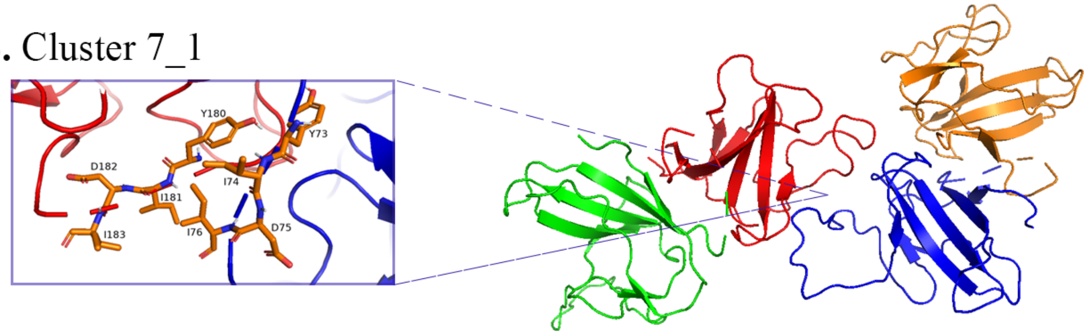


```
   126  131  136  141  146  151  156  161  166  171  176  181  186  191  196  201  206  211  216  221  226
SNAQECSLQSCTQHQPYVVDDPCPIHFYSKWYIRVGARKSAPLIELCVDEAGSKSPIQYIDIGNYTVSCLPFTINCQEPKLGSLVVRCSFYEDFLEYHDVRVVLDFI
15    21   26   31   36   41   46   51   56   61   66   71   76   81   86   91   96   101  106  111  116
SNAQECSLQSCTQHQPYVVDDPCPIHFYSKWYIRVGARKSAPLIELCVDEAGSKSPIQYIDIGNYTVSCLPFTINCQEPKLGSLVVRCSFYEDFLEYHDVRVVLDFI
   126  131  136  141  146  151  156  161  166  171  176  181  186  191  196  201  206  211  216  221  226
SNAQECSLQSCTQHQPYVVDDPCPIHFYSKWYIRVGARKSAPLIELCVDEAGSKSPIQYIDIGNYTVSCLPFTINCQEPKLGSLVVRCSFYEDFLEYHDVRVVLDFI
```

C. Cluster 8_1

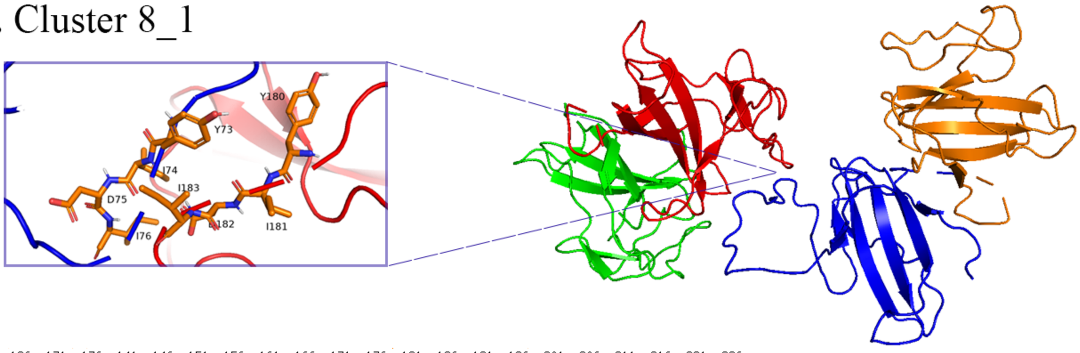


```
   126  131  136  141  146  151  156  161  166  171  176  181  186  191  196  201  206  211  216  221  226
SNAQECSLQSCTQHQPYVVDDPCPIHFYSKWYIRVGARKSAPLIELCVDEAGSKSPIQYIDIGNYTVSCLPFTINCQEPKLGSLVVRCSFYEDFLEYHDVRVVLDFI
15    21   26   31   36   41   46   51   56   61   66   71   76   81   86   91   96   101  106  111  116
SNAQECSLQSCTQHQPYVVDDPCPIHFYSKWYIRVGARKSAPLIELCVDEAGSKSPIQYIDIGNYTVSCLPFTINCQEPKLGSLVVRCSFYEDFLEYHDVRVVLDFI
```

D. Cluster 9_1

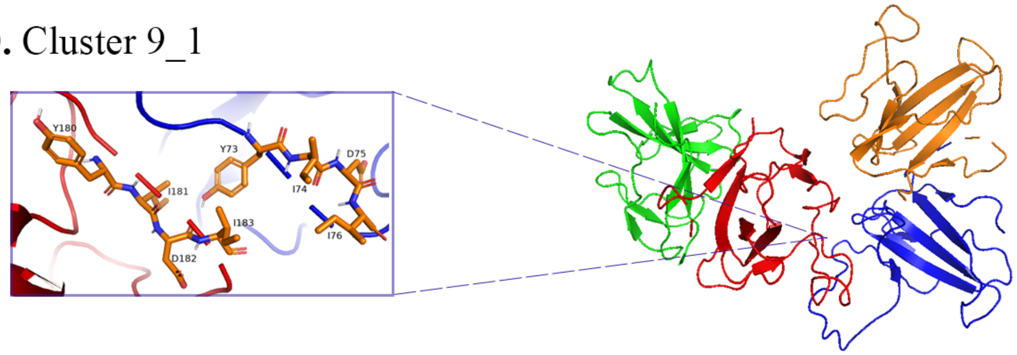


```
   126  131  136  141  146  151  156  161  166  171  176  181  186  191  196  201  206  211  216  221  226
SNAQECSLQSCTQHQPYVVDDPCPIHFYSKWYIRVGARKSAPLIELCVDEAGSKSPIQYIDIGNYTVSCLPFTINCQEPKLGSLVVRCSFYEDFLEYHDVRVVLDFI
15    21   26   31   36   41   46   51   56   61   66   71   76   81   86   91   96   101  106  111  116
SNAQECSLQSCTQHQPYVVDDPCPIHFYSKWYIRVGARKSAPLIELCVDEAGSKSPIQYIDIGNYTVSCLPFTINCQEPKLGSLVVRCSFYEDFLEYHDVRVVLDFI
   126  131  136  141  146  151  156  161  166  171  176  181  186  191  196  201  206  211  216  221  226
SNAQECSLQSCTQHQPYVVDDPCPIHFYSKWYIRVGARKSAPLIELCVDEAGSKSPIQYIDIGNYTVSCLPFTINCQEPKLGSLVVRCSFYEDFLEYHDVRVVLDFI
```

A. Cluster 10_1

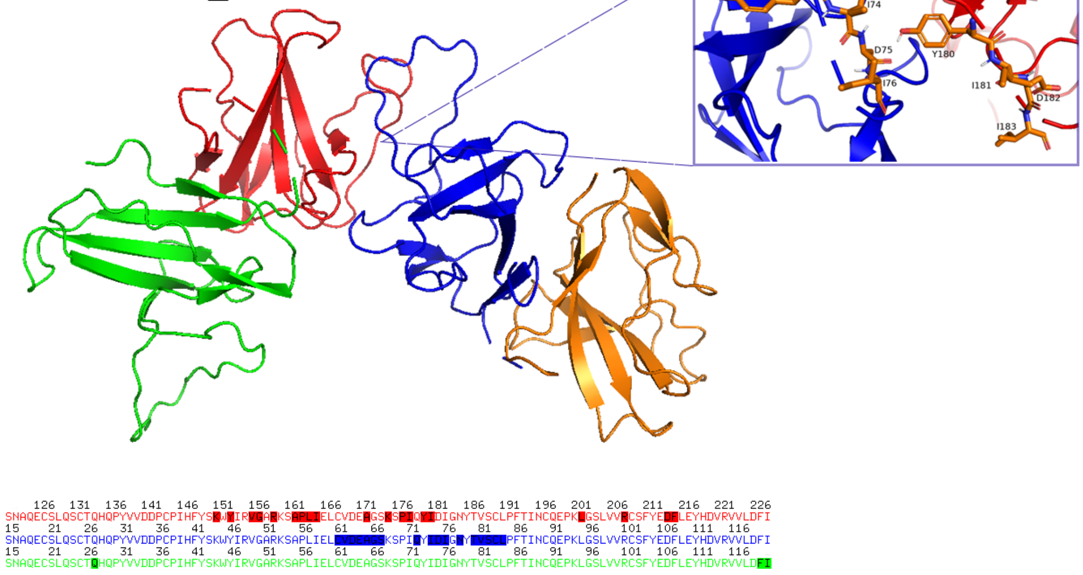

B. Cluster 11_1

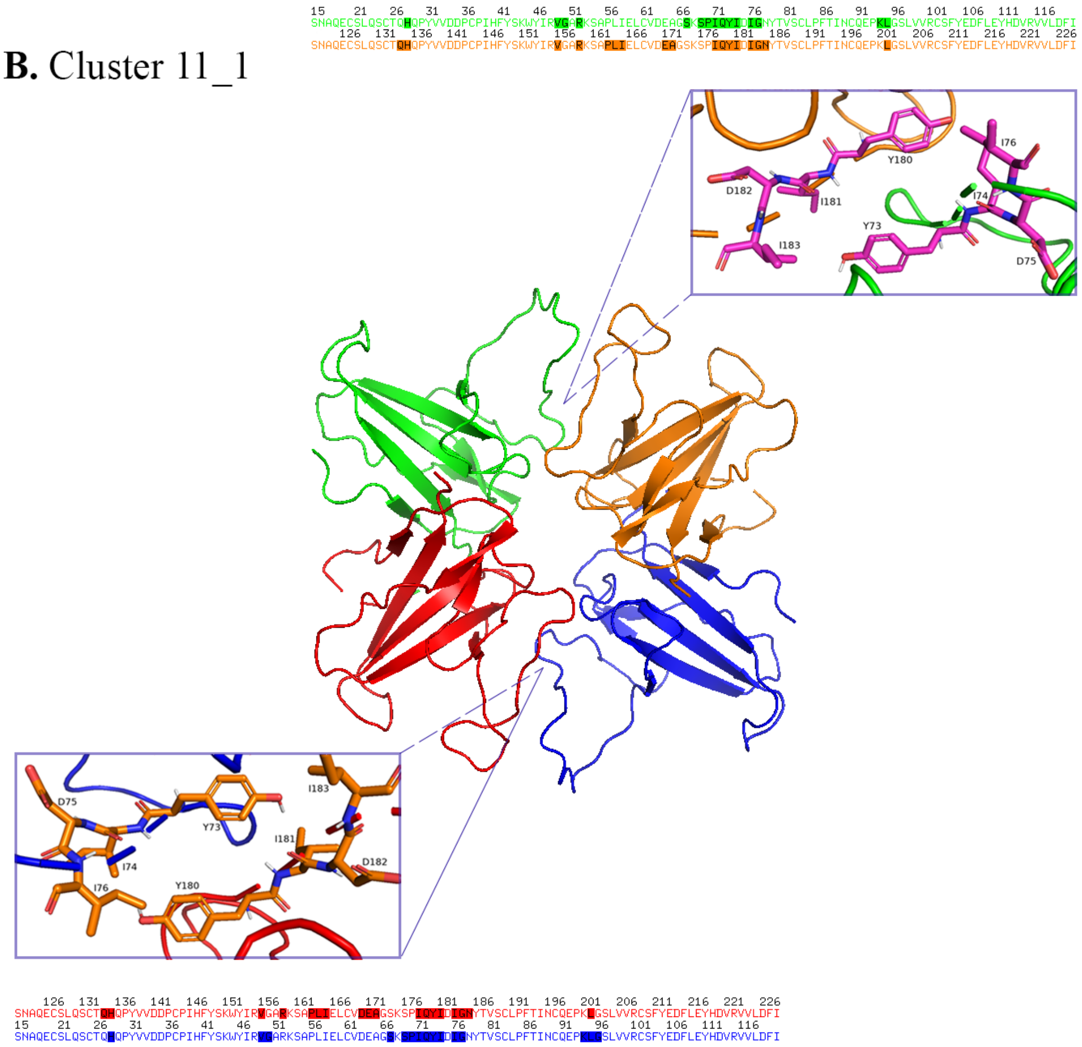

Set 2

Cluster number	3	2	4	5	1	6
HADDOCK score	-135.4 +/- 4.2	-124.9 +/- 4.3	-87.5 +/- 4.3	-75.3 +/- 14.1	-71.3 +/- 1.5	-35.3 +/- 5.5
Cluster size	37	38	10	5	79	4
RMSD	1.0 +/- 0.6	2.5 +/- 0.2	6.1 +/- 0.3	1.4 +/- 0.2	3.6 +/- 0.2	6.3 +/- 0.3
Van der Waals energy	-85.7 +/- 6.6	-80.6 +/- 4.2	-50.0 +/- 4.3	-48.0 +/- 6.8	-43.2 +/- 2.1	-24.0 +/- 2.7
Electrostatic energy	-105.4 +/- 30.1	-83.3 +/- 7.2	-51.2 +/- 4.5	-39.3 +/- 25.5	-13.5 +/- 4.5	0.2 +/- 3.6
Desolvation energy	-32.8 +/- 1.5	-33.0 +/- 2.2	-33.8 +/- 1.6	-25.2 +/- 3.1	-27.6 +/- 2.5	-19.0 +/- 0.4
Restraints violation energy	42.0 +/- 24.5	53.2 +/- 23.5	65.4 +/- 20.3	57.8 +/- 14.8	22.2 +/- 15.8	76.5 +/- 25.2
Buried Surface Area	2946.9 +/- 145.7	2532.9 +/- 108.6	1944.9 +/- 94.5	2018.1 +/- 243.8	1739.3 +/- 102.5	1082.6 +/- 71.0
Z-Score	-1.4	-1.1	0.0	0.4	0.5	1.6

Table S3. Statistics of tetramers constructed by two non-covalent interfaces defined docking.

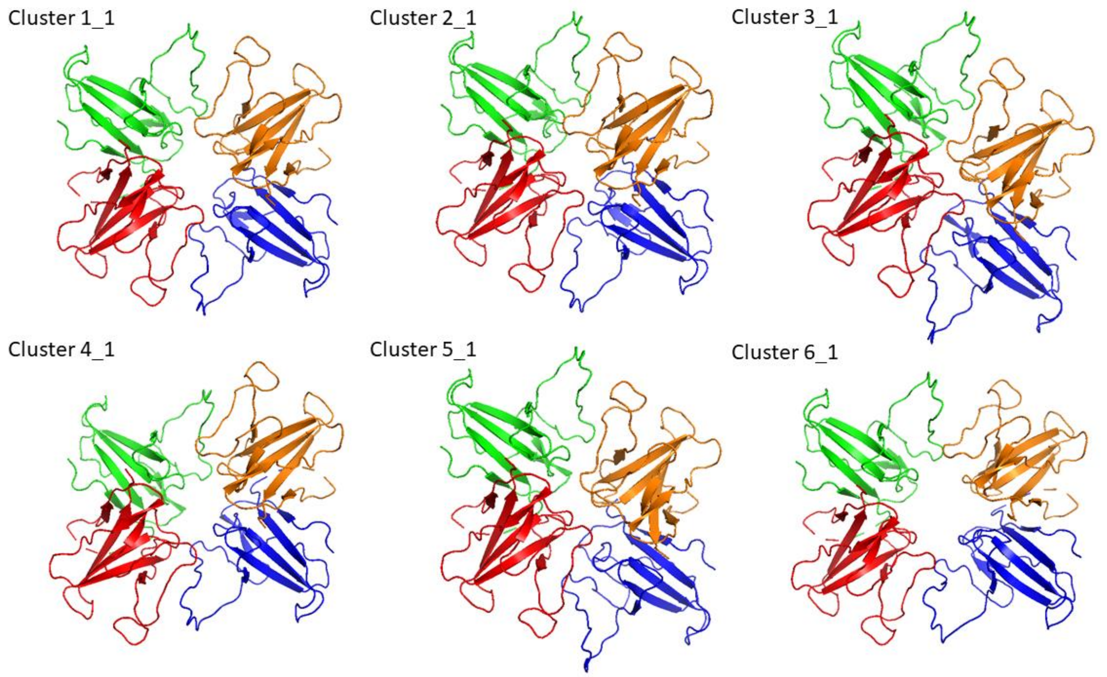

Figure S5. Two non-covalent interfaces centered by the YIDI motif bind dimers in all clusters. Cartoon representation of best clusters' structures.

A. Cluster 1_1

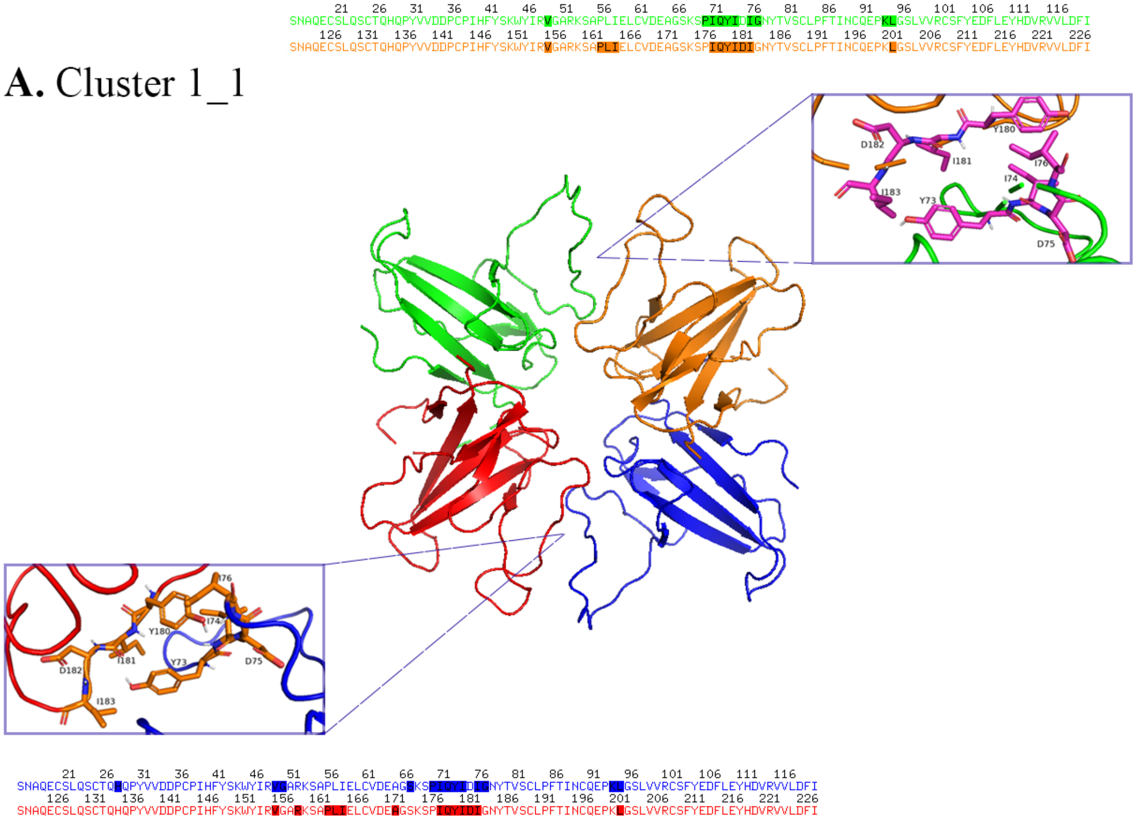

B. Cluster 2_1

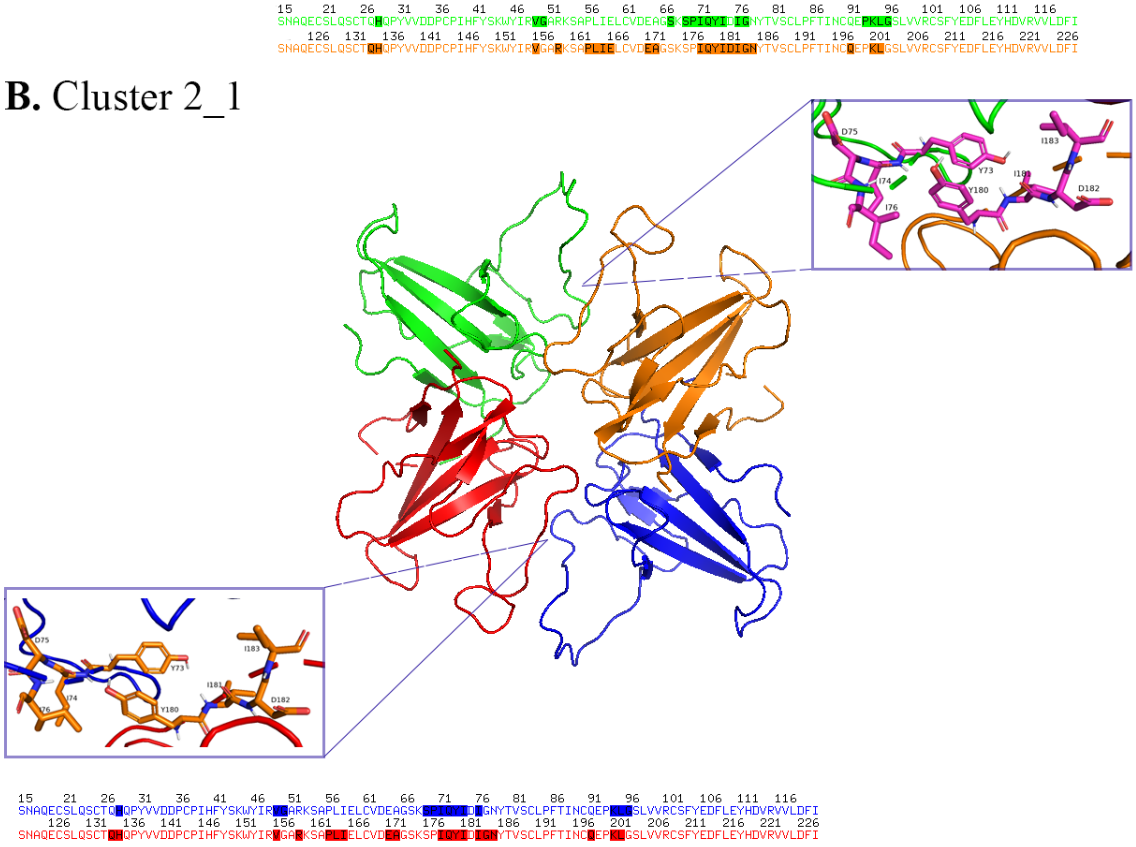

A. Cluster 3_1

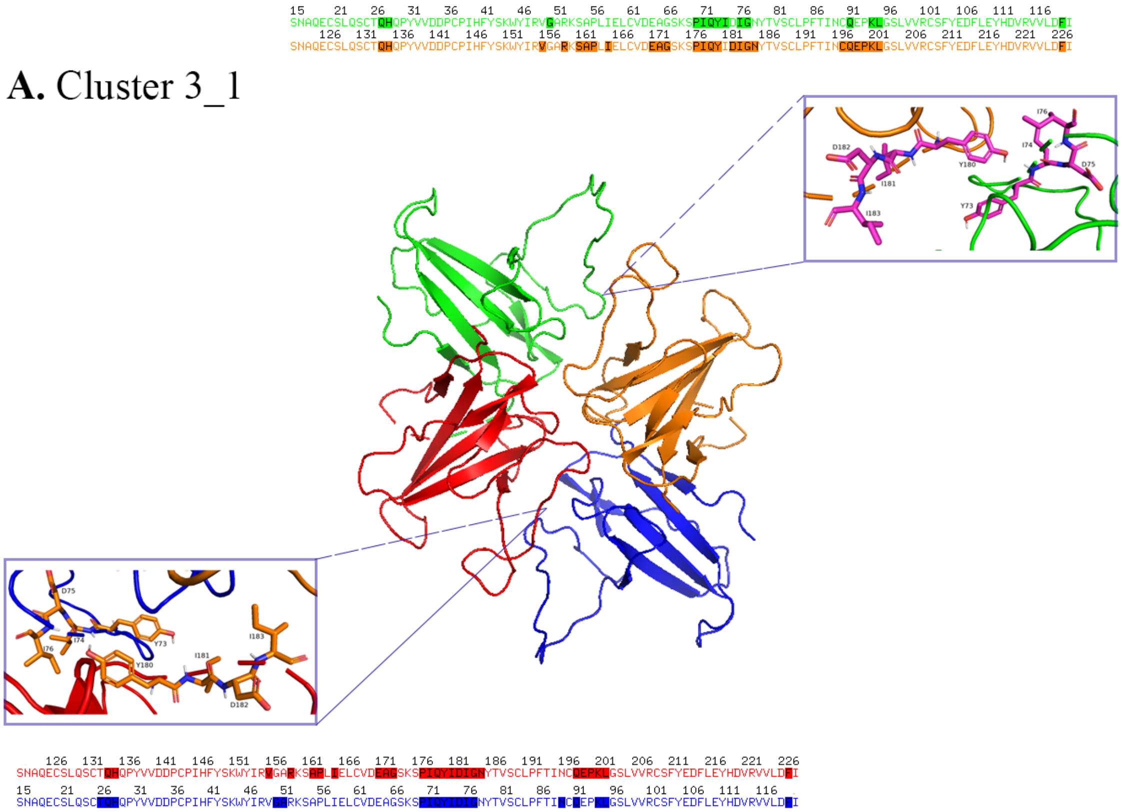

B. Cluster 4_1

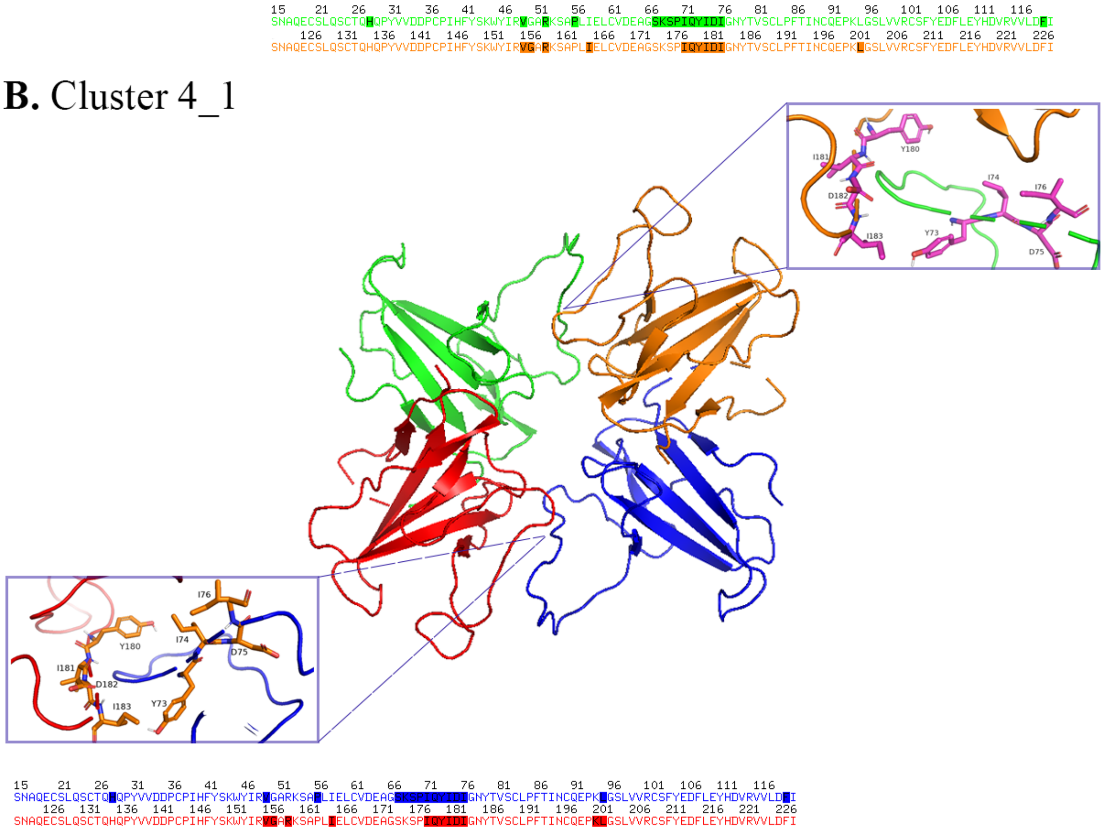

A. Cluster 5_1

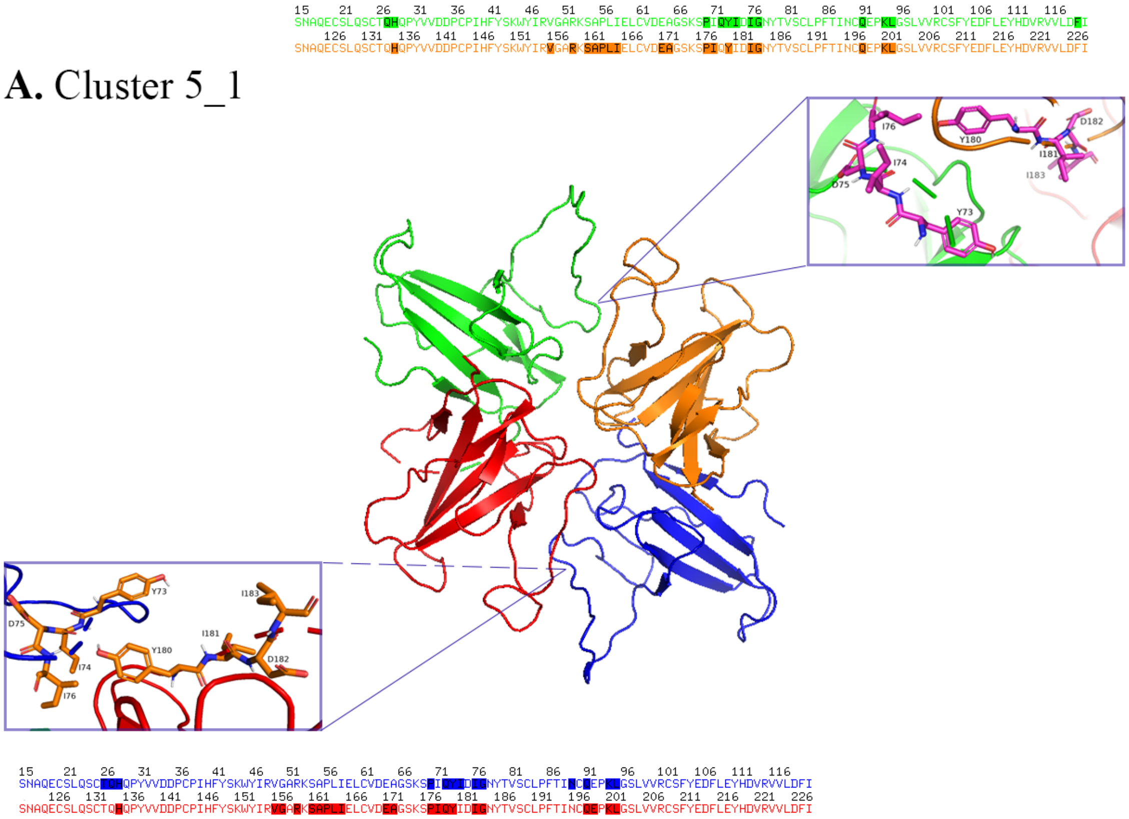

B. Cluster 6_1

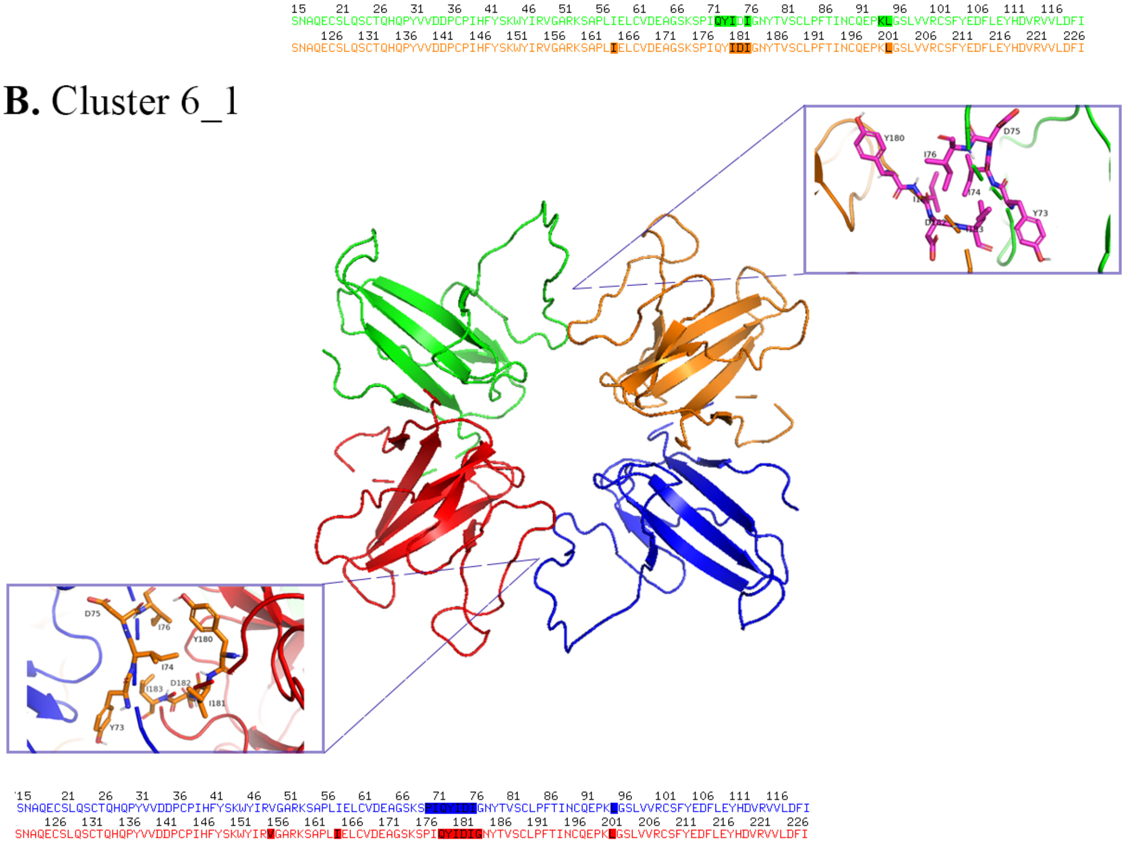

Molecular dynamics simulations

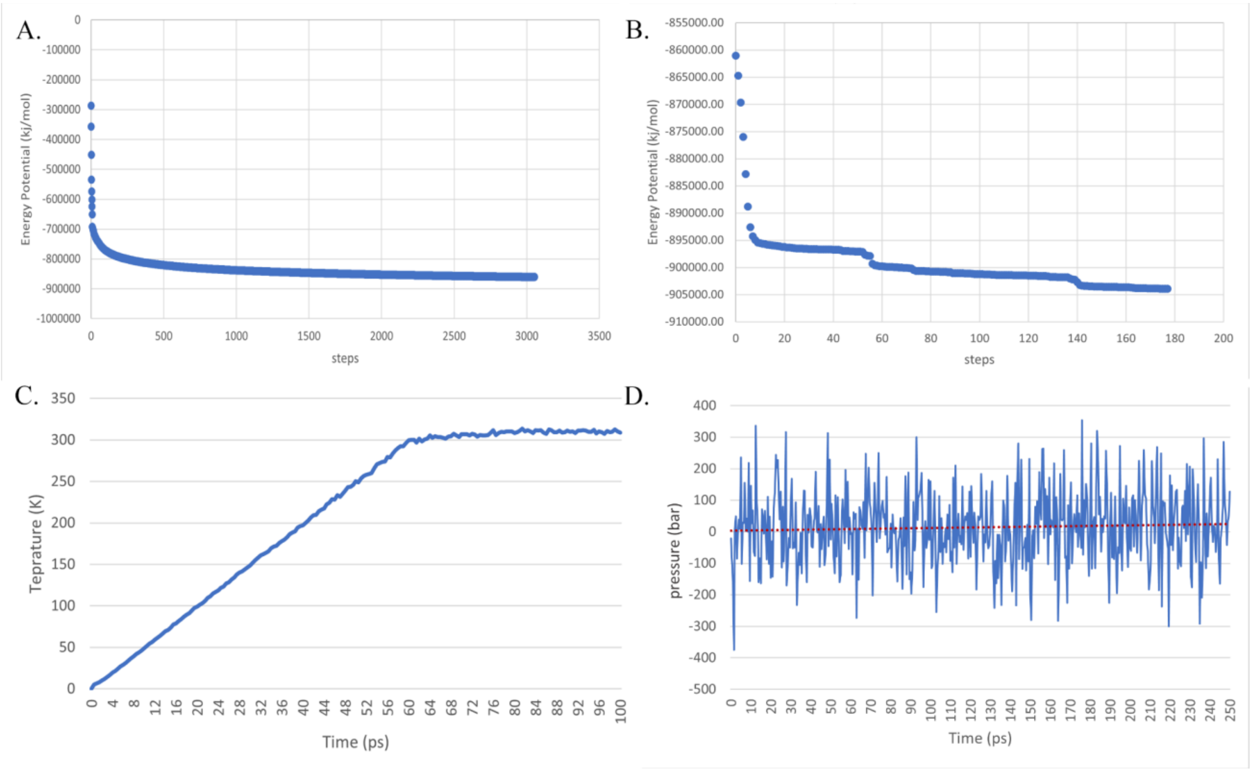

Figure S6. The trimer system preparation for MD production simulation. **(A)** steepest stem energy minimization. **(B)** conjugate gradient energy minimization. **(C)** NVT equilibration. **(D)** NPT equilibration.

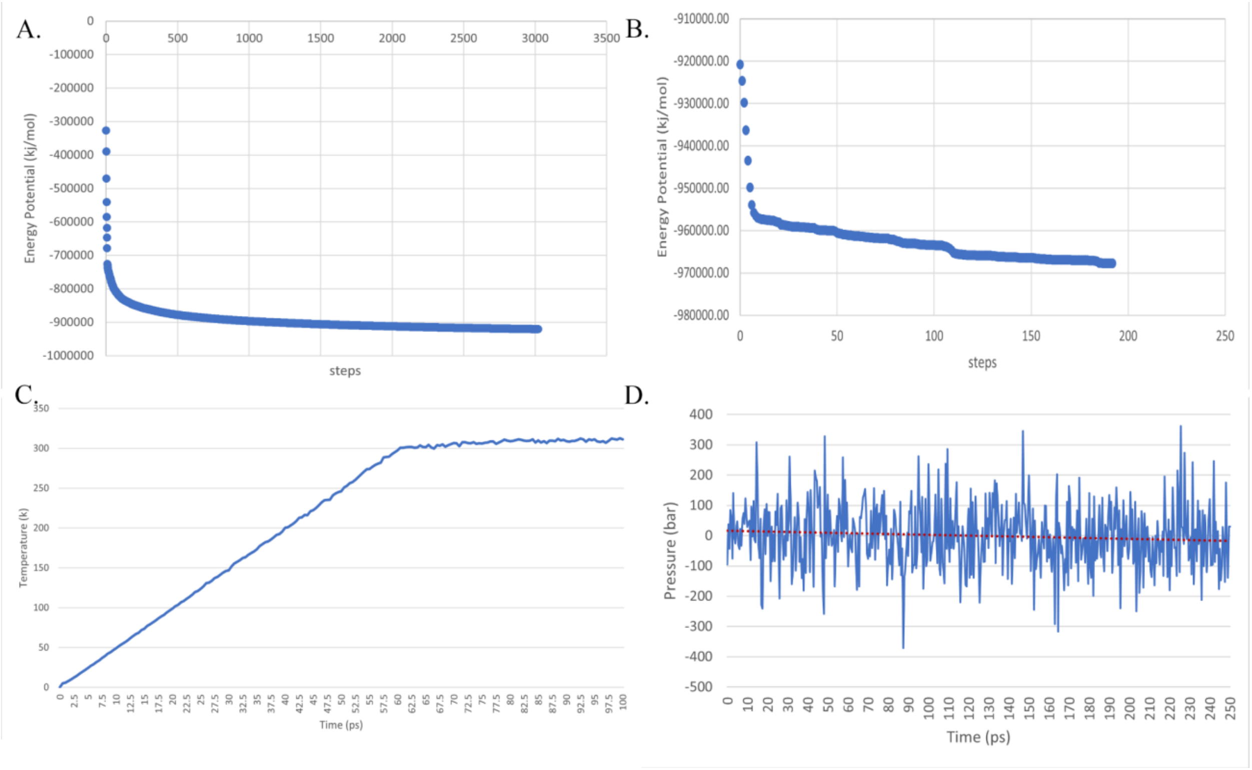

Figure S7. The tetramer system preparation for MD production simulation. **(A)** steepest stem energy minimization. **(B)** conjugate gradient energy minimization. **(C)** NVT equilibration. **(D)** NPT equilibration.

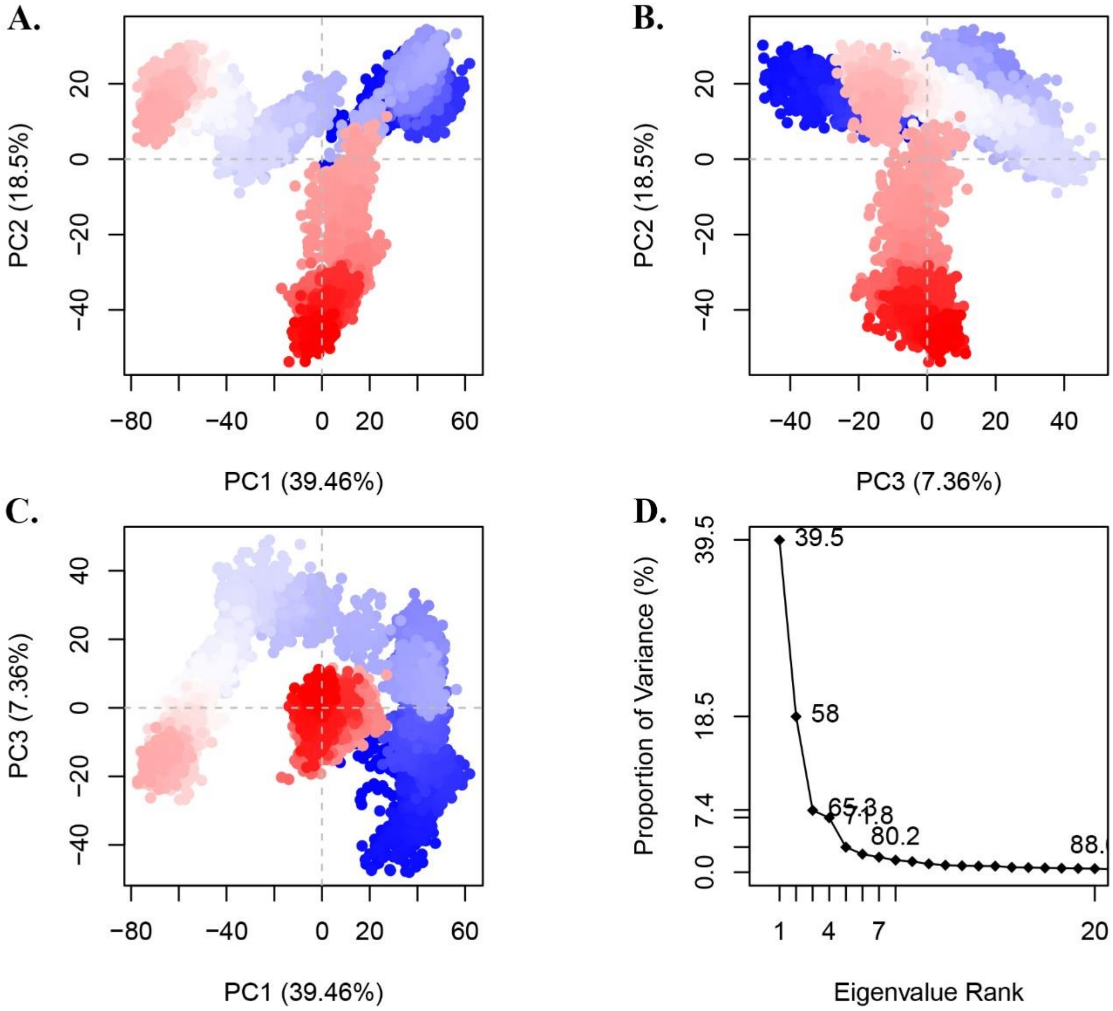

Figure S8. Projection of the tetrameric structures along PC1, PC2, and PC3. Each point represents a frame from the trajectories and the shades of red and blue represent the progression of time. **(A)** PC1-PC2. **(B)** PC2-PC3. **(C)** PC1-PC3. **(D)** scree plot.

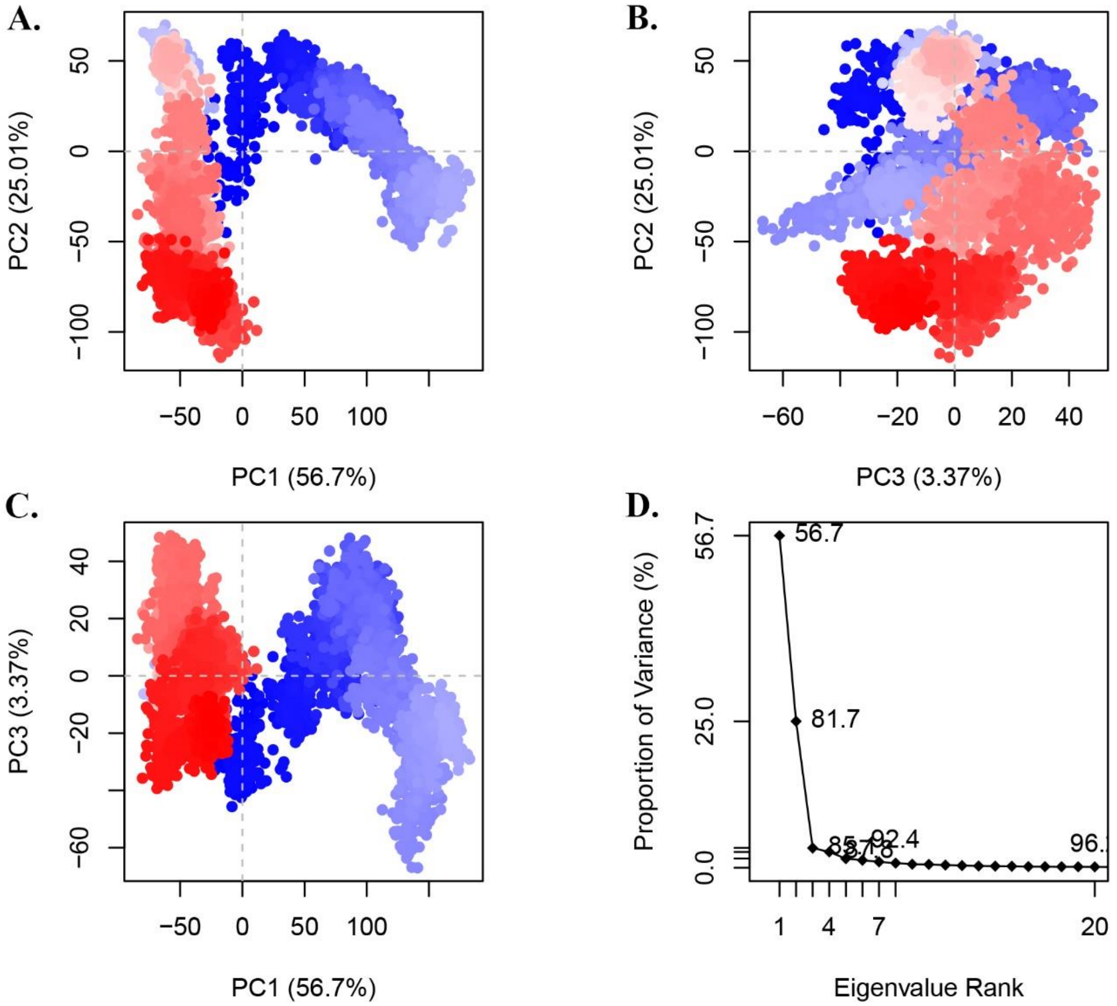

Figure S9. Projection of the trimeric structures along PC1, PC2, and PC3. Each point represents a frame from the trajectories and the shades of red and blue represent the progression of time. (**A**) PC1-PC2. **(B)** PC2-PC3. **(C)** PC1-PC3. **(D)** scree plot.

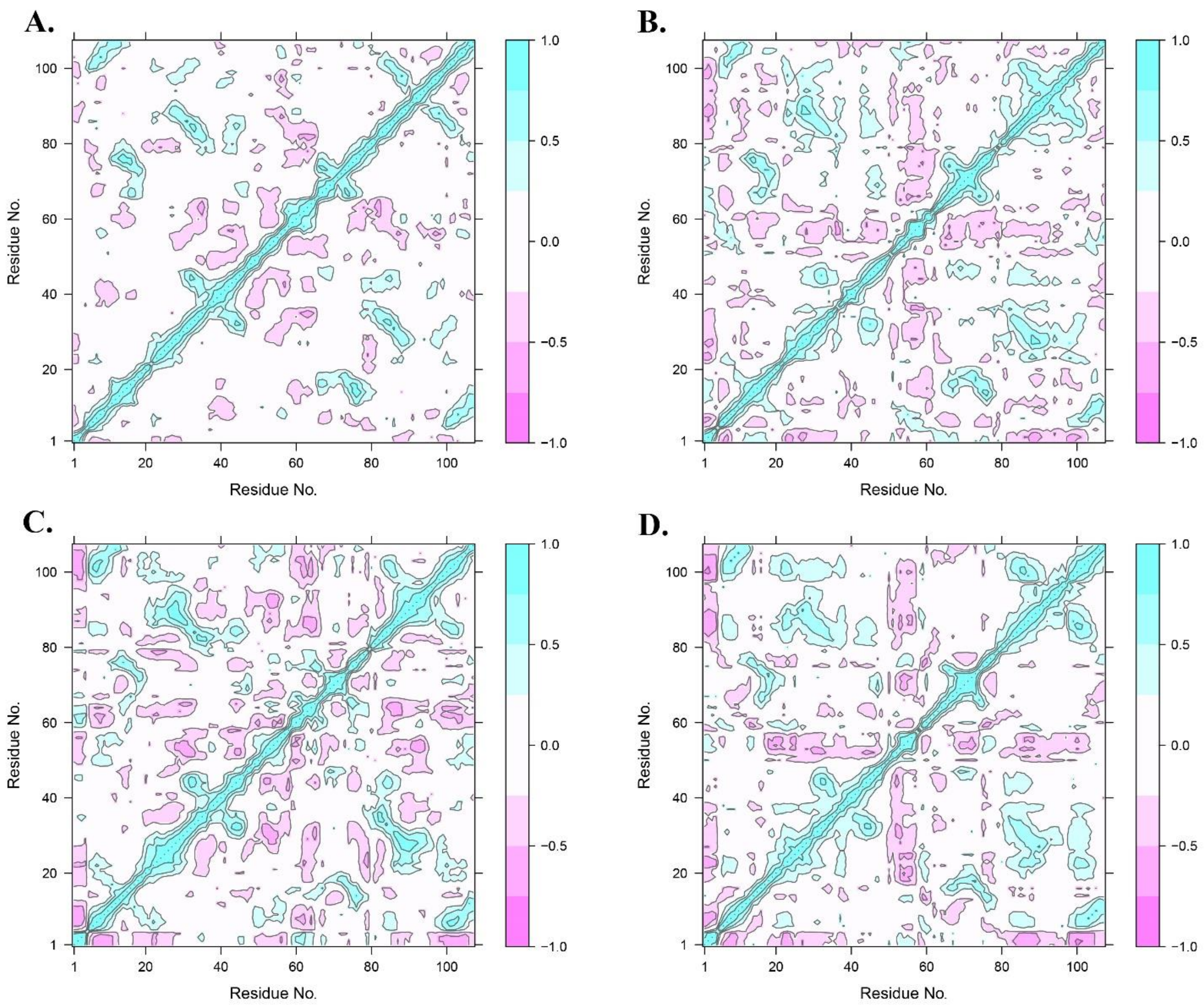

Figure S10. The monomer-wise residue cross-correlation matrices of tetramers. pink and cyan spots present the atom's correlated and anti-correlated motions, respectively. DCCM of Monomers A, B, C, and D are represented in **(A)**, **(B)**, **(C)**, and **(D)**, respectively.

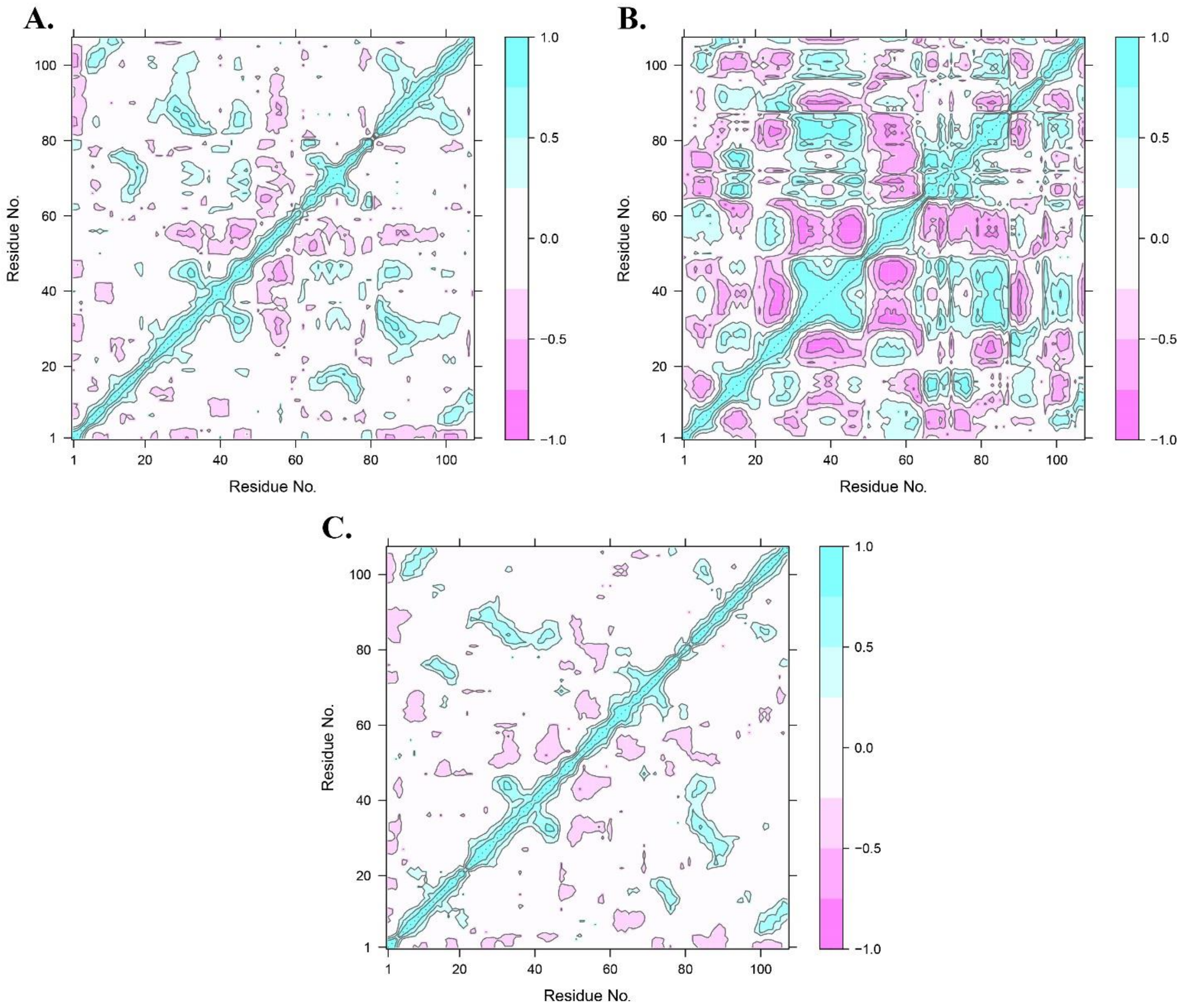

Figure S11. The monomer-wise residue cross-correlation matrices of trimers. pink and cyan spots present the atom's correlated and anti-correlated motions, respectively. DCCM of Monomers A, B, and C are represented in **(A)**, **(B)**, and **(C)** respectively.

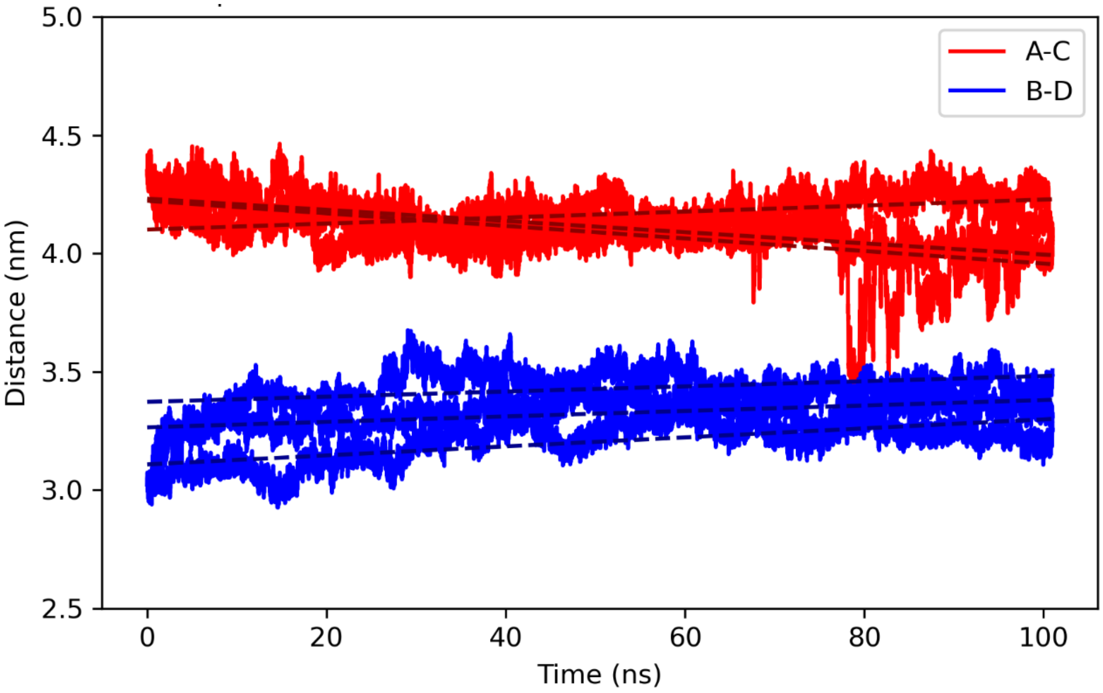

Figure S12. The Time-dependent distance between COM of chains in the tetrameric ORF8. red lines show the distance of COM of monomers A and C while the blue lines show that of monomers B and D. The dark-colored dashed lines indicate the linear trends of related trajectories.

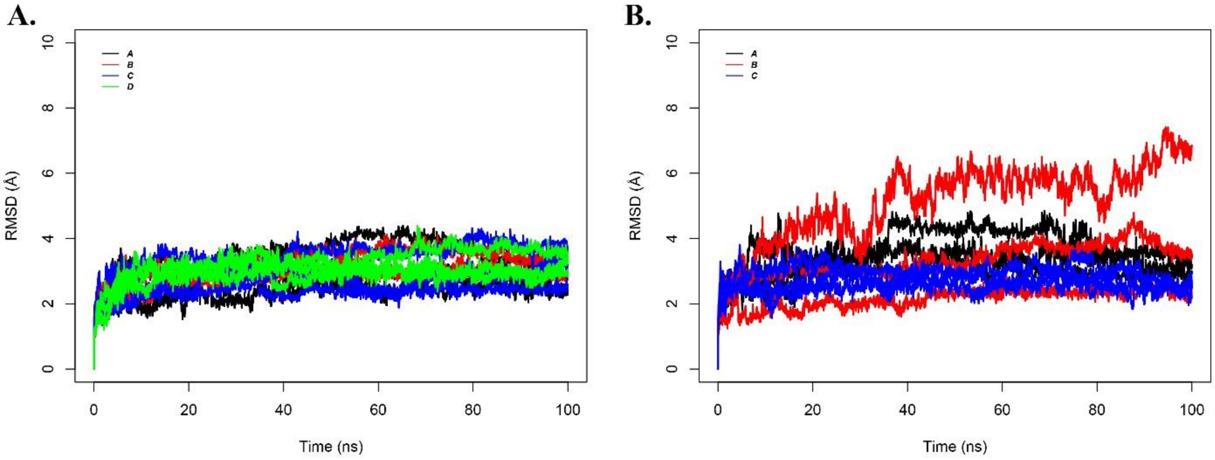

Figure S13. Subunit-wise RMSD plots of **(A)** tetramers and **(B)** trimers. Monomer B of the trimer exhibits a relatively distinct pattern.

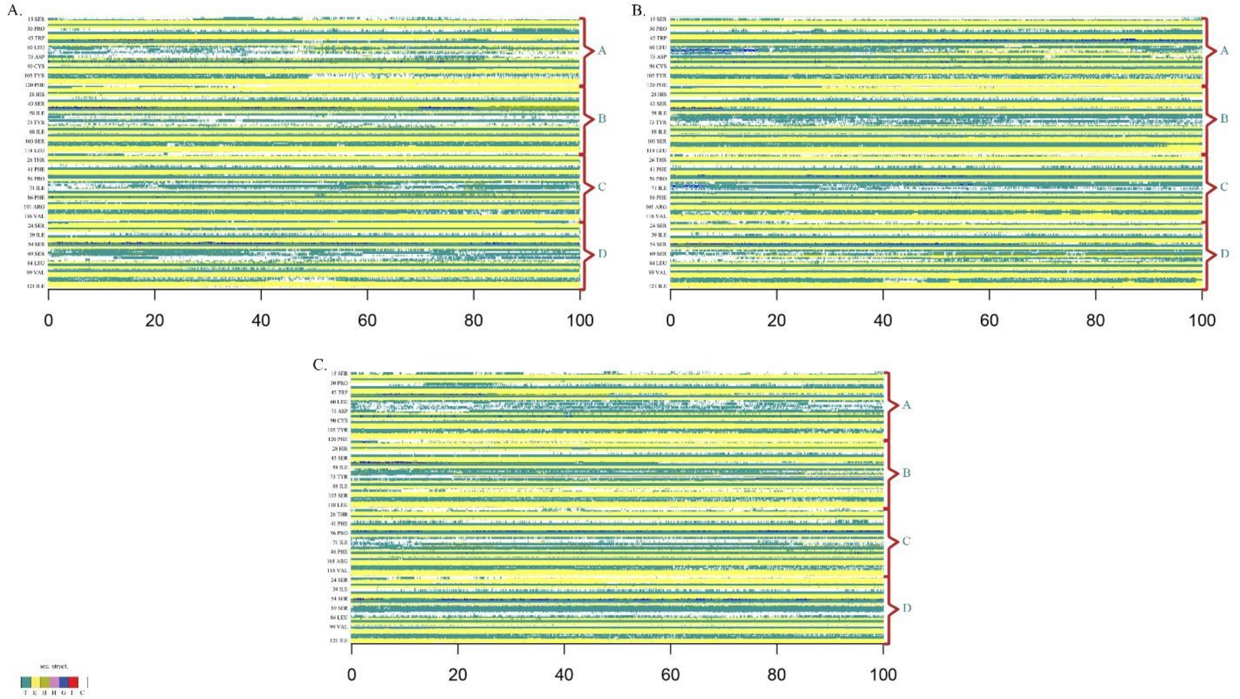

Figure S14. Secondary structure analysis of tetramer trajectories. The first, second, and third replication are shown in **(A)**, **(B)**, and **(C)**.

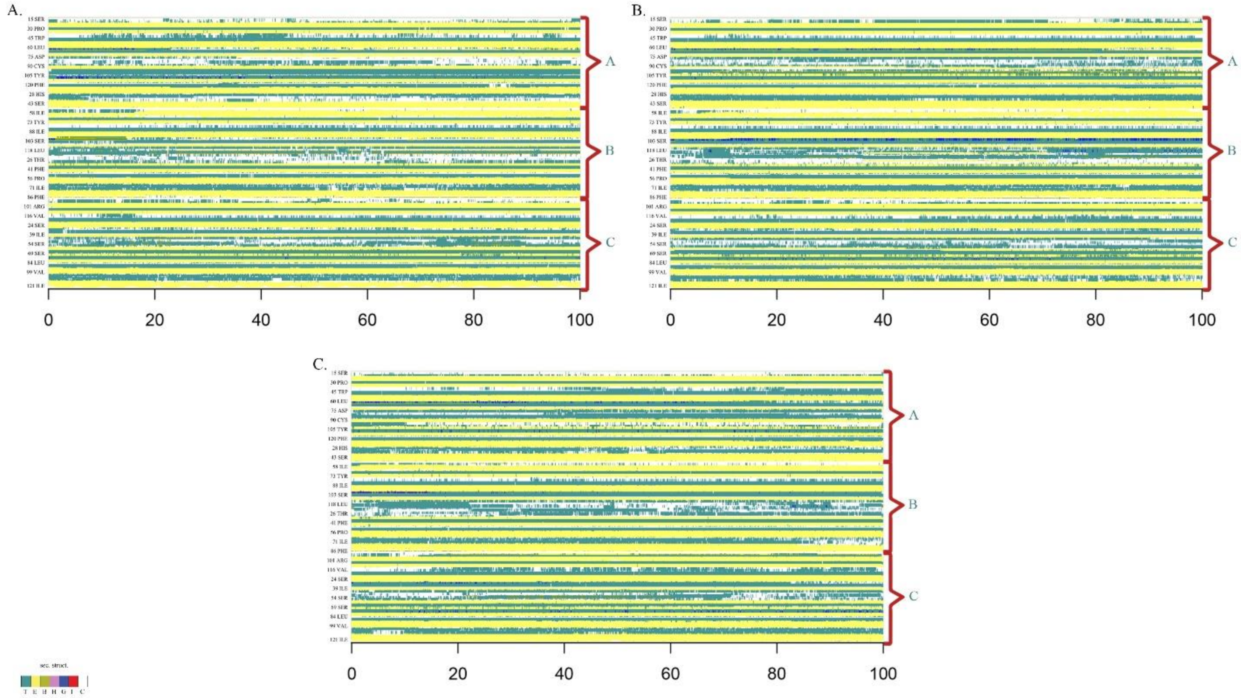

Figure S15. Secondary structure analysis of trimer trajectories. The first, second, and third replication are shown in **(A)**, **(B)**, and **(C)**.

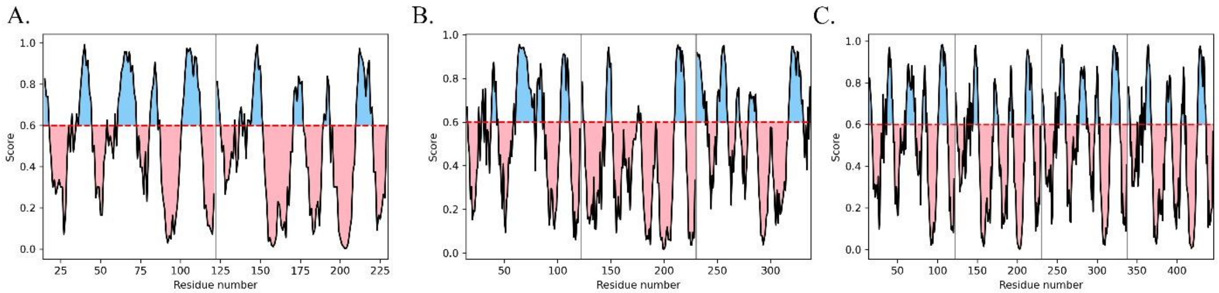

Figure S16. The per-residue protrusion Index plots of dimer, trimer, and tetramer were shown in **(A)**, **(B)**, and **(C)** respectively. The high scores in the light blue areas indicate potential B-cell epitopes.
